# Efficient and biologically relevant consensus strategy for Parkinson’s disease gene prioritization

**DOI:** 10.1186/s12920-016-0173-x

**Published:** 2016-03-09

**Authors:** Maykel Cruz-Monteagudo, Fernanda Borges, Cesar Paz-y-Miño, M. Natália D. S. Cordeiro, Irene Rebelo, Yunierkis Perez-Castillo, Aliuska Morales Helguera, Aminael Sánchez-Rodríguez, Eduardo Tejera

**Affiliations:** CIQUP/Departamento de Química e Bioquímica, Faculdade de Ciências, Universidade do Porto, Porto, 4169-007 Portugal; Instituto de Investigaciones Biomédicas (IIB), Universidad de Las Américas, 170513 Quito, Ecuador; REQUIMTE, Department of Chemistry and Biochemistry, Faculty of Sciences, University of Porto, 4169-007 Porto, Portugal; REQUIMTE, Department of Biochemistry, Faculty of Pharmacy, University of Porto, 4050-313 Porto, Portugal; Sección Físico Química y Matemáticas, Departamento de Química, Universidad Técnica Particular de Loja, San Cayetano Alto S/N, EC1101608 Loja, Ecuador; Molecular Simulation and Drug Design Group, Centro de Bioactivos Químicos (CBQ), Central University of Las Villas, Santa Clara, 54830 Cuba; Departamento de Ciencias Naturales, Universidad Técnica Particular de Loja, Calle París S/N, EC1101608 Loja, Ecuador

**Keywords:** Consensus strategy, Co-expression networks, Early recognition, Gene prioritization, Parkinson’s disease

## Abstract

**Background:**

The systemic information enclosed in microarray data encodes relevant clues to overcome the poorly understood combination of genetic and environmental factors in Parkinson’s disease (PD), which represents the major obstacle to understand its pathogenesis and to develop disease-modifying therapeutics. While several gene prioritization approaches have been proposed, none dominate over the rest. Instead, hybrid approaches seem to outperform individual approaches.

**Methods:**

A consensus strategy is proposed for PD related gene prioritization from mRNA microarray data based on the combination of three independent prioritization approaches: *Limma*, machine learning, and weighted gene co-expression networks.

**Results:**

The consensus strategy outperformed the individual approaches in terms of statistical significance, overall enrichment and early recognition ability. In addition to a significant biological relevance, the set of 50 genes prioritized exhibited an excellent early recognition ability (6 of the top 10 genes are directly associated with PD). 40 % of the prioritized genes were previously associated with PD including well-known PD related genes such as SLC18A2, TH or DRD2. Eight genes (CCNH, DLK1, PCDH8, SLIT1, DLD, PBX1, INSM1, and BMI1) were found to be significantly associated to biological process affected in PD, representing potentially novel PD biomarkers or therapeutic targets. Additionally, several metrics of standard use in chemoinformatics are proposed to evaluate the early recognition ability of gene prioritization tools.

**Conclusions:**

The proposed consensus strategy represents an efficient and biologically relevant approach for gene prioritization tasks providing a valuable decision-making tool for the study of PD pathogenesis and the development of disease-modifying PD therapeutics.

**Electronic supplementary material:**

The online version of this article (doi:10.1186/s12920-016-0173-x) contains supplementary material, which is available to authorized users.

## Background

Parkinson’s disease (PD) is the second most common neurodegenerative disorder (ND). The present annual cost of health care for patients with PD is estimated to exceed $ 5.6 billion just in the US. With the rapid increase in worldwide life expectancy, the prevalence of PD is expected to double by 2030 [1–3].

Dopamine replacement drugs remains the principal and most effective treatment for PD [4]. However, as the disease progresses, their efficacy diminishes and fails to address the degeneration observed in other brain areas [5–7]. Ultimately, disease-modifying treatments are needed that address both the motor and nonmotor symptoms of PD.

Currently the most important diagnostic marker of PD is limited to the presence of motor disturbances. Unfortunately, due to overlap of symptoms with other neurodegenerative disorders, misdiagnosis is common. Moreover, motor deficits allowing clinical diagnosis generally appear when 50–60 % of dopaminergic neurons in the *substantia nigra* (SN) are already lost, limiting the effectiveness of potential neuroprotective therapies [8].

In addition to motor symptoms, non-motor symptoms including autonomic dysfunction, depression, olfactory deficit, cognitive disturbances and sleep abnormalities have been related to PD [9]. This mixture of apparently unrelated symptoms and physiological disorders highlight that PD is a multi-causal disorder. Thus, to identify new targets and biomarkers for PD becomes critical for the early diagnosis of this medical condition and for the development of disease-modifying therapies.

In this sense, the systemic picture of gene expression information enclosed in mRNA microarrays experiments encodes relevant clues on the pathogenesis, biomarkers or therapeutics targets for a disease state, but requires of approaches able to unravel it through the accurate prioritization of those disease relevant genes [10]. Several bioinformatics approaches have been reported for this task including those based on differential gene expression [11], gene co-expression networks [12] or machine learning (ML) approaches [13].

Each approach has particular theoretical foundations determining relative advantages and limitations. It is well known that the consensus use of multiple and independent pieces of information increases the reliability of a decision-making process [14]. So, the hybridization of conceptually different approaches can provide prioritization tools with enhanced efficiency [15]. Specifically, such novel hybrid approaches have not been applied yet to PD relevant genes prioritization nor even to neurodegenerative disorders [12]. In this work we propose a consensus strategy for PD relevant genes prioritization based on the integration of several approaches including linear models for microarray data (*Limma*), machine learning, and co-expression networks. Since only a few candidates can usually be considered for further validation experiments, particular emphasis is made in the early recognition ability prioritization tools.

One problem benchmarking the early recognition ability of prioritization approaches in bioinformatics is the lack of statistically sound metrics for this task [16]. Other related areas such as chemoinformatics have standardized procedures to evaluate an analogous problem to gene prioritization, the virtual screening [17]. Here we propose for the first time the use of such early recognition metrics to evaluate the performance of gene prioritization approaches. Hence, besides to identify an enriched set of PD related genes we propose a consensus strategy for gene prioritization with proved enrichment efficiency and biological relevance, as well as a statistically founded approach to evaluate the early recognition ability of gene prioritization tools.

## Methods

### Microarrays data

Experimental microarray data comparing healthy control (HC) and Parkinson’s disease (PD) samples were obtained analyzing the Gene Expression Omnibus (GEO) [18]. Table 1 shows the GEO data sources, references, and sample distribution used in the study. Only studies on *substantia nigra* were considered. So, eight samples collected from *frontal gyru*s were removed from GSE8397.

**Table 1:** Microarray data details

Code	Platform	Sample	Ref.
GSE20292^a^	GPL96	11(PD); 18(HC)	[45, 53]
GSE7621	GPL570	16(PD); 9(HC)	[46]
GSE20333	GPL201	6(PD); 6(HC)	[97]
GSE8397^b^	GPL96	31(PD); 16(HC)	[47]

^a^Three samples with outlier nature removed after cross-platform normalization

^b^Eight samples collected from *frontal gyrus* removed

It is important to highlight that the *substantia nigra* is the region of the brain that shows the greatest loss of dopaminergic neurons in human PD patients. This induce a serious bias that we will term the “dopamine bias”. This bias induce a serious risk of overestimation of the enrichment ability of a prioritization strategy based on samples coming from the *substantia nigra*. At the same time, it is also true that dopamine-related process are intrinsically implicated in the pathogenesis of PD. So, we need to check not only which prioritized gene is “dopamine-related”, but also whether such gene is associated or not with PD. This critical issue will be considered along all the analysis conducted and properly discussed in the following sections of the manuscript.

Each microarray was processed as follows: public data was extracted and processed using *GEOquery* package in Bioconductor [19]. After individual microarrays analysis, the first step in cross-platform microarray analysis is to combine the different probes. For this task the *entrez gene* was used as identifier in order to obtain the common space across all platforms [20–22]. We mapped the arrays probes of each independent studies to the respective *entrez gene* ID through manual observation and also using the updated manufacturers annotation information (using R-packages: *hgu133a.db*, *hgu133plus2.db* and *hgfocus.db* [23–25]) for all platforms.

Only genes common to all platforms (8477 genes) were used in the subsequent analysis. Genes with more than one probe in individual microarray/studies were combined using the row with the highest mean intensity value applying the *collapseRows* and *intersect* functions implemented in the *WGCNA* package [26, 27]. A second normalization was performed in order to re-scale the intensity and remove cross-platform batch effects using the *Combat* function of the *SVA* package [28]. From the initial set of 29 samples in GSE20292 three samples with outlier nature were removed after cross-platform normalization. Finally a subset of 102 samples (59 PD and 43 HC) remained for further analysis.

### Differential gene expression analysis

The identification of genes with statistically different expression between HC and PD groups was performed using *lmFit* from *Limma* R-Package [29]. The basic statistic used for significance analysis was the moderated t-statistic after adjustment with the Benjamini and Hochberg’s method to control the false discovery rate (“fdr” adjusted *p*-values) [30].

### Machine learning analysis

The ML analysis was conducted over a cross-platform normalized microarray data including 8477 common genes for 102 samples. The full data was split up into training and test sets, as part of the validation scheme [31]. Approximately 25 % of the samples were randomly assigned to the “Test Set” by using the *Create a Subset/Random (Stratified) Sampling* option implemented in STATISTICA 8.0 [32]. Details on the final distribution of the 102 samples can be assessed on Additional file 1: Table S1. Normalized expression values of the 8477 common genes for each of the 102 samples, sample and study identifiers, disease factor (PD or HC), as well as the distribution of training and test samples are provided as supplementary information Additional file 2.

The full vector of 8477 normalized gene expression values was reduced to 500 genes with maximal relevance for the disease factor by means of the minimal redundancy maximal relevance (mRMR) software [33]. Details of the reduced gene set by using the mRMR software are provided in the supplementary information. Then, the reduced vector was subject to an independent process of feature selection relying on eleven different ranking feature selection algorithms implemented on WEKA 3.7.11 [34]. See the full list of attribute evaluators in the supplementary information. Additionally, the reduced vector was subject to a wrapper subset selection using as attribute evaluators only those ML classifiers including a subset feature selection stage implemented on WEKA 3.7.11.

### Weighted gene co-expression network construction and analysis

The full set of 8477 common genes was used for weighted genes co-expression network (WGCN) construction in each group using the *WGCNA* package [27]. In this study, we set the β parameter variation to 6, following the scale-free topology criterion proposed by Zhang and Horvath using the *pickSoftThreshold* function in *WGCNA* [35]. Once defined the adjacency matrix for each group (HC and PD), the corresponding co-expression matrices (CoHC and CoPD) were obtained.

### Modular analysis

The modules were detected using the *Dynamic Tree Cut* algorithm [36] by using the *cutreeDynamic* function implemented in the *WGCNA* package. Here, the deep split was set to 3, the cutting height to the 99th percentile and the joining heights on the dendograms were set to the maximum. The node connectivity (*k*) and the node intramodular connectivity (*k*_*intra*_) were calculated for each module as described in [37].

### Statistical significance

The gene ontology (GO) and diseases enrichment analysis were performed using *DAVID bioinformatics resource v6.7* [38], exploiting the well know Gene Ontology Annotation (GOA) [39] and Genetic Association (GAD) [40] databases. The ToppCluster tool for the combined enrichment analysis [41] was used to provide network representations of individual and common terms. The statistical significance of the respective enrichment analyses was accessed by using FDR criteria with *p*-value < 0.05 as cut-off.

The statistical significance of each genes set prioritized as relevant for PD was assessed as proposed by Chen et al. [42, 43]. Detailed information on the application of this test is provided in the supplementary information. Additionally, a bootstrap random sampling experiment was implemented in *R* as proposed by [42, 43] and performed to test the probability of randomly selecting the same number of known PD related genes in the prioritized genes sets. The Wilcoxon signed rank test was used as significance test.

### Enrichment and early recognition

Several enrichment metrics have been proposed in the chemoinformatics literature to measure the enrichment ability of a VS protocol [17]. However, despite being bioinformatic’s gene prioritization and chemoinformatic’s virtual screening essentially the same problem, this type of enrichment analysis has not been applied in bioinformatics. In this work, we use some of the most extended metrics to estimate the enrichment ability of the gene prioritization strategies proposed. The overall enrichment metrics used here include the area under the accumulation curve (*AUAC*); the area under the ROC curve (*ROC*); and the enrichment factor (*EF*) evaluated at the top 1 %/5 %/10 %/20 % of the ranked list. At the same time, the early recognition metrics used were the robust initial enhancement (*RIE*) and the Boltzmann-enhanced discrimination of ROC (*BEDROC*) evaluated at the top 1 %/5 %/10 %/20 % of the ranked list [17]. The calculation of both classic and early recognition enrichment metrics was conducted by using the perl script *Cresset_VS* [44].

## Results and discussion

### *Limma* based gene prioritization

First, the background of 8477 genes provided by the 102 samples of HC and PD patients was processed with *Limma*. The goal here is to identify those single genes significantly differentiated between HC and PD samples and so, potentially associated with PD. This procedure identified a set of 134 genes with an “fdr” adjusted *p-values* < 0.05, each of which was considered to be significantly differentiated on PD patients. Details on this set of genes are reported as supplementary information. The results of the disease enrichment analysis are shown in Table 2. The number of genes associated with PD and included in GAD provides evidence of a statistically significant association of the selected set of genes with PD (*p*-value = 0.0271).

**Table 2:** Disease enrichment analysis on the Genetic Association Database of a set of 134 genes prioritized for PD by using *Limma*

GAD Term	*p-Value*	Hits Sample	Total Sample	Hits Background	Total Background
bipolar disorder	0.0030	7	39	96	2459
schizophrenia	0.0034	11	39	249	2459
alcohol abuse	0.0227	4	39	40	2459
Parkinson’s disease	0.0271	6	39	112	2459
delinquent behavior violent behavior	0.0307	2	39	2	2459
schizophrenia; opium abuse	0.0307	2	39	2	2459
alcoholism	0.0346	4	39	47	2459
nicotine dependence smoking behavior	0.0457	2	39	3	2459
impulsivity	0.0457	2	39	3	2459
bipolar affective disorder; unipolar affective disorder	0.0457	2	39	3	2459
personality traits	0.0480	3	39	23	2459

*Hits Sample*: Number of genes selected by *Limma* that are asociated with the disease condition; *Total Sample*: Number of genes selected by *Limma*; *Hits Background*: Number of genes in the background that are asociated with the disease condition; *Total Background*: Number of genes in the background

It is important to note that the GAD database only covers 29 % of the top 134 genes prioritized using an FDR corrected *p*-value < 0.05 as significance cutoff. Similarly, the OMIM database have only a coverage of just 25 %. Accordingly, the ranking provided by the disease enrichment analysis must be used as reference instead of a exact criterion of the degree of association of the prioritized genes set with the disease. Consequently, the information in Table 2 can be only used to support the statistically significant association between the top 134 genes prioritized by *Limma* and PD.

However, if we use an uncorrected *p*-value < 0.5 as a significance cutoff instead of the FDR corrected p-value, the set of prioritized genes increases notably to 1016 genes with a non statistically significant association with PD (data not shown). Such a radical change supports the choice in this work to use FDR corrected instead of uncorrected p-values. It could be explained by the well-knwon ability of the FDR correction to minimize the number of false negatives [30] which minimize the lost of PD related genes and consequently, increasing the enrichment of the gene set selected by using this criterion.

The full list of the top 1016 genes prioritized are provided as a suplementary information (see Additional file 5). In this list we can find several genes reported in previous transcriptome analysis based on similar samples [45–51], some using the same micrarray data used in our work. Even so, it is hard to know the real degree of overlapping between our genes and those reported in these works because not every paper reports the full list of significantly differentiated genes. Moreover, in these works several dissimilar processing strategies were applied which impose and additional degree of difficulty on the comparison across these and our study.

If we look for example to the works reported in [47, 48, 51, 52], the degree of overlaping between the genes lists reported is extremely low. Actually, no common genes were found between the four studies and the maximal overlapping between two studies were two common genes (LRRFIP1 and MDH1) between [5] and [6]. Such a minimal degree of overlapping could be atributed to the diversity of tissues, samples or methodological approaches applied on each independent study. However, when the unique set of 243 genes extracted from the combination of the genes sets reported in [47, 48, 51, 52] is compared with our genes prioritized with *Limma*, a significantly higher degree of overlaping is found. Specifically, a 4.92 % of overlapping (50 common genes) is found considering the top 1016 genes (using the uncorrected *p*-value < 0.05 as a significance cutoff); 8.21 % of overlapping (11 common genes) considering the top 134 genes (using FDR corrected *p*-value < 0.05); and 6.49 % of overlapping (39 common genes) considering the top 608 genes (using FDR corrected *p*-value < 0.25). The last top fraction of 608 genes using a cutoff of 0.25 for FDR corrected *p*-values was also included in the comparison since such a cutoff is widely used in this type of prioritizations [47–50, 53]. One should expect a higher degree of overlapping for larger gene sets. However, as described, the higher degree of overlapping was found in the top 134 genes prioritized by using FDR corrected p-values. Again, the ability of the FDR correction to minimize the number of false negatives can be the explanation to this unexpected observation.

Other genes known to be associated with PD such as TH, SLC18A2, NR4A2, DDC and SLC6A3 can be found in our *Limma* prioritization. Interestingly, compared with these genes, SNCA exhibited a lower significance. An statistically significant differenced expression of SNCA is considered mandatory for clinical diagnosis of classical PD [8, 48]. In this prioritization we noted this differencial expression (see supplementary information), but just using as a cutoff an adjusted *p*-value < 0.25, in agreement with previous studies [47–50, 53]. On the other hand, a reduction in dopamine markers as well as the the presence of α-synuclein–positive Lewy bodies in *substantia nigra* are not exclusive of PD [8, 54]. Therefore it is not surprising that the consensus approach prioritized other genes before SNCA.

A different scenario emerges from the GO enrichment analysis of biological proceses. From this analysis, the overall information extracted is that although the set of genes prioritized by *Limma* do not fully match with known genes associated with PD, the biological processes involving these genes are well known to be implicated in the pathogenia of PD. The GO terms, description, and the FDR corrrected *p*-values corresponding to the top 11 statistically significant biological process identified from the set of 134 genes are provided in Table 3. Details on the full list of biological process associated to this gene set can be accessed in the suplementary information (see Additional file 5).

**Table 3:** GO terms, description, and the FDR corrrected p-values corresponding to the statistically significant biological process identified from 134 genes prioritized by *Limma*

GO terms	Description	*p*-value (FDR)
GO:0006576	biogenic amine metabolic process	3,3E-04
GO:0042401	biogenic amine biosynthetic process	3,7E-04
GO:0034311	diol metabolic process	8,2E-04
GO:0009712	catechol metabolic process	8,2E-04
GO:0006584	catecholamine metabolic process	8,2E-04
GO:0018958	phenol metabolic process	9,8E-04
GO:0042423	catecholamine biosynthetic process	3,2E-03
GO:0042398	cellular amino acid derivative biosynthetic process	1,4E-02
GO:0042416	dopamine biosynthetic process	2,3E-02
GO:0006575	cellular amino acid derivative metabolic process	2,9E-02
GO:0042417	dopamine metabolic process	4,4E-02

The information provided in Table 3 clearly reveals an enrichment in dopamine and neurotransmition process. Although the key role of dopamine metabolism in PD is well known [6], the reduction of dopamine synthesis or simply changes in the metabolism of the dopamine are not exclusive of PD. Such effect in other neurodegenerative disorders or even aging has been recently discussed [51]. Additionally, we can not rule out that the enrichment observed in dopamine process could be a possible consequence of a particular degradation in the *substantia nigra* or even a combined factor for neuronal loss in this particularly sensible tissue [48, 50]. Obviously, is not possible to isolate these effects without aditional experimental data. We also found (although with FDR corrected *p*-values < 0.05) other biological process well stablished in PD such as oxidative fosforilation and energetic metabolism [46–49, 53] (see details in the supplementary information). The lack of statistical significance of these process is obviously a direct consequence of the reduction of the gene set comming from the use FDR corrrected p-values as cutoff. Actually, when the entire set of 1016 genes (using uncorrected *p*-values) is subject to the same GO enrichment analysis, these processes become significantly more enriched than dopaminergic processes. The details on the GO enrichment analysis are provided as supplementary information (see Additional file 5). This also indicates that even when a bias toward dopamine metabolism exist, additional information relevant to PD is enclosed in the microarray data used. As discussed later, the consensus strategy actually favor the inclusion of such non dopamine related process.

Finally, another important finding to mention is that the transcriptional coactivator PPARGC1A (PGC-1α) was not found to be significantly differenciated in our study, even when it is a master regulator of mitochondrial biogenesis and oxidative metabolism [48, 50]. In this sense, it is important to note that these studies applied different methodologies so to find this gene as not significantly differentiated is a perfectly possible scenario. The fact that only one of the four studies used in this work reported this gene as diferentially expressed support this observation. Finally, even when PPARGC1A was not found in our study, several genes were found to be direct interactors, and biological process directly related with this gene are clearly present in our prioritized genes. It is elaborated further based on the results shown by the functional interaction network of the set of 50 genes finally prioritized.

### Machine learning based gene prioritization

For the ML based gene prioritization process, the full vector of 8477 normalized gene expression values was first reduced to 500 genes with maximal relevance for the disease factor (see the full list in the Additional file 3). This set of 500 genes comprises the 91 % of the 134 genes prioritized by *Limma*. This indicates that this initial gene set used as input for feature selection and further ML modeling conserves almost the same information prioritized by *Limma*. Then, the reduced vector was subject to an independent process of feature selection as previously depicted in Methods section. Once ranked the 500 relevant genes by the respective attribute selection method, each gene is scored according to their mean rank position across the eleven attribute evaluators by applying a desirability function [55]. The corresponding gene relevance score *d*(*Rank*_*i*_) is defined as:

1 $$ \begin{array}{cc}\hfill d\left( Ran{k}_i\right)=\frac{Ran{k}_i-1}{1- Ran{k}_{max}}\hfill & \hfill 0\le\ d\left( Ran{k}_i\right)\le 1\hfill \end{array} $$

Here *Rank*_*i*_ denotes the rank position assigned to the gene *i* by the attribute evaluator while *Rank*_*max*_ is determined by the number of genes to rank and corresponds to the worst possible rank position (500th). Finally, the overall relevance score for a gene *i* deduced from the consensus ranking analysis *D*(*Rank*_*i*_) is computed as the arithmetic mean of the *d*(*Rank*_*i*_) values across all the attribute evaluators applied.

Next, the 500 genes previously identified were also subject to a wrapper subset selection as described in Methods section. The relevance of the subset of genes selected is deduced from the accuracy of the respective classifier. So, we only considered as relevant those subset of genes coming from classifiers exhibiting values of accuracy, sensitivity and specificity over 0.6 on training and validation sets. Table 4 provides details of the predictive performance of the thirteen ML classifiers. Considering the classification performance we can assert that based on the set of genes identified by each ML algorithm it is possible to classify the disease status of our microarray samples with a confidence ranging from 75 to 83 % (see Table 4). The sets of genes selected by the respective classifiers are provided in Additional file 1: Table S2.

**Table 4:** Classification performance of the ML classification algorithms used to identify PD relevant sets of genes

ML Classification Algorithm	Training set			LOO CV			5-Fold CV			Test set		
	*Acc.*	*Se.*	*Sp.*	*Acc.*	*Se.*	*Sp.*	*Acc.*	*Se.*	*Sp.*	*Acc.*	*Se.*	*Sp.*
*functions.SimpleLogistic*	1.000	1.000	1.000	0.827	0.860	0.781	0.827	0.814	0.844	0.704	0.750	0.636
*rules.MODLEM*	1.000	1.000	1.000	0.813	0.837	0.781	0.760	0.767	0.750	0.778	0.750	0.818
*rules.PART*	0.987	0.977	1.000	0.653	0.674	0.625	0.747	0.721	0.781	0.741	0.750	0.727
*trees.ADTree*	1.000	1.000	1.000	0.853	0.860	0.844	0.787	0.721	0.875	0.741	0.750	0.727
*trees.BFTree*	0.973	1.000	0.938	0.853	0.884	0.813	0.747	0.744	0.750	0.741	0.750	0.727
*trees.FT*	1.000	1.000	1.000	0.800	0.837	0.750	0.867	0.884	0.844	0.741	0.813	0.636
*trees.LADTree*	1.000	1.000	1.000	0.840	0.884	0.781	0.827	0.814	0.844	0.889	0.875	0.909
*trees.LMT*	1.000	1.000	1.000	0.813	0.860	0.750	0.773	0.767	0.781	0.741	0.813	0.636
*trees.SimpleCart*	0.973	1.000	0.938	0.827	0.837	0.813	0.747	0.721	0.781	0.741	0.750	0.727
*meta.AdaBoostM1*	1.000	1.000	1.000	0.840	0.884	0.781	0.880	0.907	0.844	0.926	1.000	0.818
*meta.AttributeSelectedClassifier*	0.960	0.977	0.938	0.680	0.721	0.625	0.760	0.767	0.750	0.852	0.875	0.818
*meta.ClassificationViaRegression*	0.960	0.977	0.938	0.813	0.814	0.813	0.733	0.698	0.781	0.815	0.938	0.636
*meta.Decorate*	1.000	1.000	1.000	0.893	0.860	0.938	0.867	0.837	0.906	0.963	1.000	0.909
*AVERAGE*	*0.989*	*0.995*	*0.981*	*0.808*	*0.832*	*0.777*	*0.794*	*0.782*	*0.810*	*0.798*	*0.832*	*0.748*

*Acc.* = accuracy or overall classification rate; *Se.* = sensitivity or true positives rate (% of PD samples correctly classified); *Sp.* = specificity or true negatives rate (% of HC samples correctly classified)

*functions.SimpleLogistic*: Classifier for building linear logistic regression models [104]; *rules.MODLEM*: Class for building and using a MODLEM algorithm to induce rule set for classification [105]; *rules.PART*: Class for generating a PART decision list [106]; *trees.ADTree*: Class for generating an alternating decision tree [107]; *trees.BFTree*: Class for building a best-first decision tree classifier [108]; *trees.FT*: Classifier for building ‘Functional trees’, which are classification trees that could have logistic regression functions at the inner nodes and/or leaves [109]; *trees.LADTree*: Class for generating a multi-class alternating decision tree using the LogitBoost strategy [110]; *trees.LMT*: Classifier for building ‘logistic model trees’, which are classification trees with logistic regression functions at the leaves [104, 111]; *trees. SimpleCart*: Class implementing a classification and regression tree with minimal cost-complexity pruning [112]; *meta.AdaBoostM1*: Metaclassifier class for boosting a nominal class classifier using the Adaboost M1 method [113]; *meta.AttributeSelectedClassifier*: Metaclassifier class where dimensionality of training and test data is reduced by attribute selection before being passed on to a classifier http://weka.sourceforge.net/doc.dev/weka/classifiers/meta/AttributeSelectedClassifier.html; *meta.ClassificationViaRegression*: Metaclassifier class for doing classification using regression methods [114]; *meta.Decorate*: Meta-learner for building diverse ensembles of classifiers by using specially constructed artificial training examples [115, 116]

Again, by applying a desirability function is possible to score the relevance of the respective gene according to the number of valid classifiers including the gene *i* and so, considering it as relevant. The corresponding gene relevance score based on the consensus classifier analysis *d*(*Class*_*i*_) ranges between 0 (only one valid classifier includes the gene) and 1 (the gene is considered relevant by all the valid classifiers) and is defined as:

2 $$ \begin{array}{cc}\hfill d\left( Clas{s}_i\right)=\frac{Nre{l}_i-1}{N_{Class}-1}\ \hfill & \hfill 0\le\ d\left( Clas{s}_i\right)\le 1\hfill \end{array} $$

Here *Nrel*_*i*_ denotes the number of valid classifiers including the gene *i* while *N*_*Class*_ indicates the number of valid classifiers.

The final subset of relevant genes proposed by the ML prioritization strategy is determined by 168 unique genes forming the union of the subsets of genes identified by the valid classifiers. Finally, the absolute relevance of each gene (*MLrel*_*i*_) is estimated by considering its respective *D*(*Rank*_*i*_) and *d*(*Class*_*i*_) scores and quantified as the corresponding arithmetic mean. Details on this set of genes are reported as supplementary information (see Additional file 4).

The final result is a list of 168 unique genes (see Additional file 5) with proved capability of discriminating PD from HC samples, and sorted according to their consensus merit (*MLrel*_*i*_). This ML set was subject to a disease enrichment analysis, providing evidence of a statistically significant association of the selected genes with PD, placing PD 2nd in the list, with *p*-value = 0.0367. However, none of the biological process involved in this set of genes was statistically significant. It is important to note that ML methods are focused on maximizing the correct classification rate. So, contrary to standard prioritization methods based on gene expression data, the set of genes identified with ML favor the relevance for the disease state instead the gene connectivity information or the biological background. Accordingly, it is unlikely that the final gene list prioritized by ML methods provide statistically significant enrichments of biological processes or pathways.

### Gene co-expression network modules prioritization

Using the *Dynamic Tree Cut* method, 9 and 16 modules were identified in CoHC and CoPD, respectively. Details on the connectivity profile of both co-expression networks are provided in Table 5.

**Table 5:** Connectivity, differential expression and machine learning data used as criteria for module prioritization

Healthy Control (HC) Modules										
Module	*n*	*<k>*	*<kintra>*	*<logPD-logHC>*	*nML*	*Merit_ML*	*nLimma*	*Merit_Limma*	*nML-Limma*	*Merit_ML-Limma*
HC_01	123	12.04	1.38	−0.021	3	1.23	1	0.51	1	1.23
HC_02	349	34.57	7.29	−0.061	6	0.87	13	2.36	4	1.73
HC_03	1057	19.04	8.85	0.011	4	0.19	2	0.12	2	0.29
HC_04	169	17.02	2.59	−0.002	1	0.30	0	0.00	0	0.00
HC_05	347	9.23	2.59	0.165	2	0.29	1	0.18	1	0.44
HC_06	74	8.26	0.73	0.005	0	0.00	0	0.00	0	0.00
HC_07	290	14.81	5.19	0.073	4	0.70	6	1.31	1	0.52
HC_08	251	10.94	2.05	0.030	11	2.21	10	2.52	5	3.02
HC_09	2	1.15	0.00	0.022	0	0.00	0	0.00	0	0.00
HC_10	37	15.32	1.48	0.043	1	1.36	0	0.00	0	0.00
HC_11	91	10.95	1.23	0.048	3	1.66	0	0.00	0	0.00
HC_12	61	23.65	3.85	0.028	2	1.65	0	0.00	0	0.00
HC_13	164	10.23	1.79	0.007	3	0.92	1	0.39	1	0.92
HC_14	71	8.33	0.81	−0.001	0	0.00	0	0.00	0	0.00
HC_15	2120	49.53	36.69	−0.062	82	1.95	97	2.89	40	2.86
HC_16	3271	22.06	14.66	−0.064	46	0.71	3	0.06	1	0.05
Parkinson’s Disease (PD) Modules										
PD_01	603	286.30	70.52	0.022	6	0.50	1	0.10	1	0.25
PD_02	1437	262.21	150.85	−0.126	69	2.42	103	4.53	42	4.42
PD_03	133	210.12	13.36	0.035	1	0.38	0	0.00	0	0.00
PD_04	161	284.83	22.96	0.089	4	1.25	3	1.18	2	1.88
PD_05	789	231.70	62.45	−0.025	5	0.32	1	0.08	0	0.00
PD_06	468	238.37	38.64	0.132	3	0.32	0	0.00	0	0.00
PD_07	494	316.82	58.43	0.103	24	2.45	19	2.43	8	2.45
PD_08	213	218.15	28.17	−0.033	4	0.95	2	0.59	1	0.71
PD_09	4179	333.39	247.08	−0.047	52	0.63	5	0.08	2	0.07

*n*: number of genes in the module*; <k>*: average node degree; *<k*_*intra*_ *>*: intra-modular average node degree; *<logPD-logHC>*: module average differential of the log transformed average expression of a gene *i* across PD samples and healthy control samples; *n*_*ML*_: number of genes identified by ML analysis included in the module; *n*_*Limma*_: number of genes identified by *Limma* analysis included in the module; *n*_*ML-Limma*_: number of common genes identified by both ML and *Limma* analyses included in the module; *Merit_ML* = (*n*_*ML*_/168)/(*N*/8477): merit assigned to the module based on *n*_*ML*_, the total number of genes identified by ML analysis (168), *N*, and the total number of background genes (8477); *Merit_Limma* = (*n*_*Limma*_/134)/(*N*/8477): merit assigned to the module based on *n*_*Limma*_, the total number of genes identified by *Limma* analysis (134), *N*, and the total number of background genes (8477); *Merit_ML-Limma* = (*n*_*ML-Limma*_/56)/(*N*/8477): merit assigned to the module based on *n*_*ML-Limma*_, the total number of common genes identified by both ML and *Limma* analyses (56), *N*, and the total number of background genes (8477)

Based on the connectivity information it should be possible to identify those modules enriched with hub genes [56, 57]. In this sense, relatively high values of the modules average node (gene) degree (*<k>*) as well as the average intramodular node degree (*<k*_*intra*_*>*) can act as relevant indicators of modules potentially enriched with hub genes. From the connectivity information four potentially PD relevant modules are identiffied. PD_07, PD_01, and PD_04 exhibit particularly high values of *<k>* while modules PD_02, and PD_01 show significantly high values of *<k*_*intra*_*>*. Among these four modules PD_07 stands out as the module with the highest overall connectivity but with barely high intramodular connectivity. On the other hand PD_02 exhibits a significant but inverse profile.

**Fig. 1:**
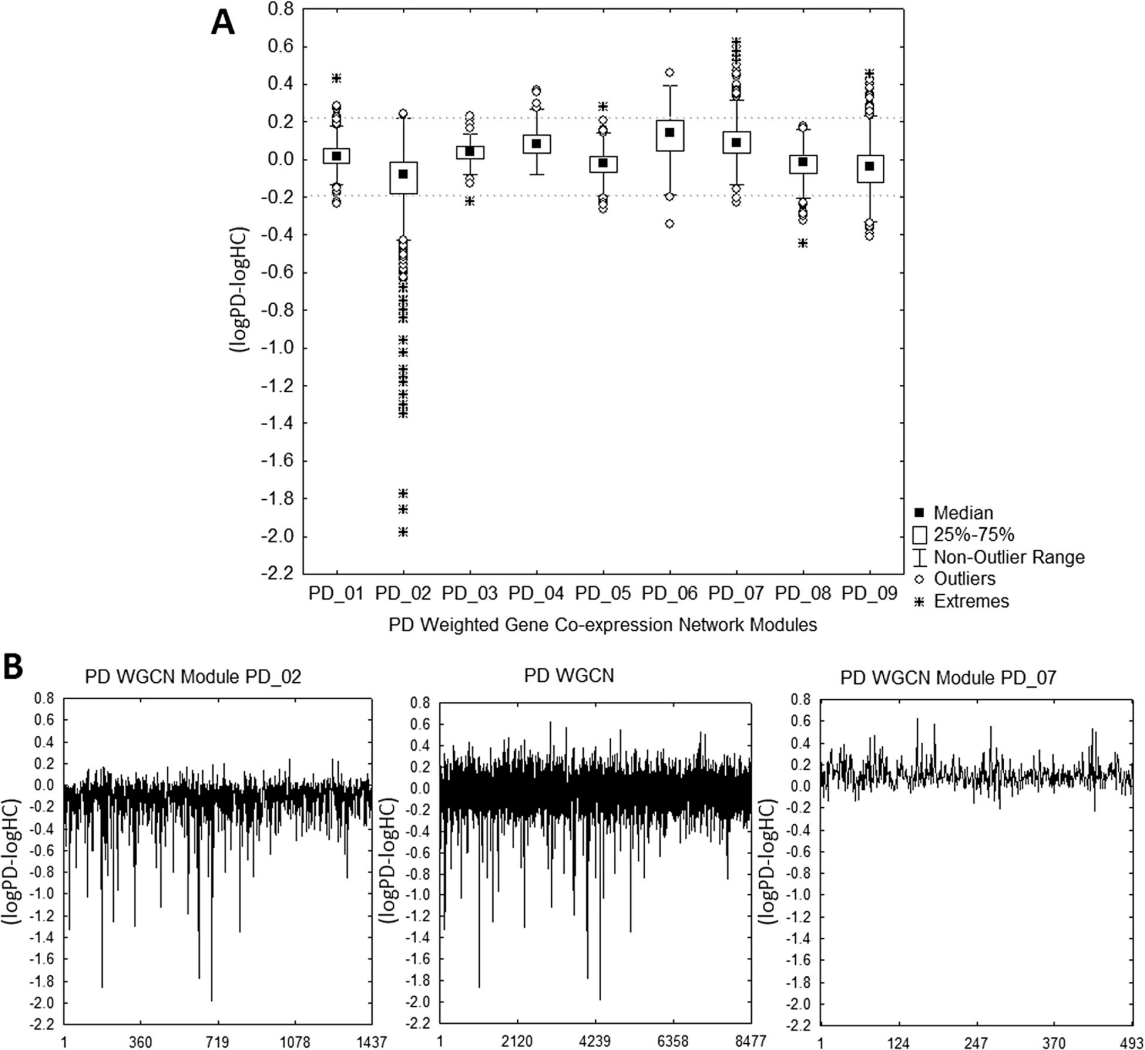
**a** Box plot of the differential average expression of genes across PD and healthy control samples (*logPD-logHC*) for genes conforming the nine PD WGCN modules. **b** Line plots of *logPD-logHC* for all the 8477 genes used to construct the global PD WGCN (*center*), 1437 genes in the predominantly underexpressed PD WGCN module PD_02 (*left*), and 494 genes in the predominantly overexpressed PD WGCN module PD_07 (*right*)

A solid decision can’t be made on the only basis of the connectivity information. So, additional information needs to be considered. For this we focused on the differential of the log transformed average expression of a gene *i* across PD samples and HC samples (*logPD-logHC*). The goal here is to identify modules enclosing genes significantly associated with PD and involved in common biological process that are central in PD [58]. Based on the average *logPD-logHC* value (see Table 5), PD_02 stands out as a significantly underexpressed module while PD_07 toguether with PD_06 are the most overexpressed modules. However, only PD_02 and PD_07 should be selected. From Fig. 1a it is clear that although PD_06 exhibit a slightly higher average *logPD-logHC* value, a significant amount of genes with outlier and extreme behaviour are only present in PD_07. From Fig. 1b it is possible to visually confirm that most of the underexpressed genes in the background (center) belongs to PD_02 (left) while most of the overexpressed genes belongs to PD_07 (right).

It is well known that the consensus use of multiple and independent pieces of information increases the reliability of a decision-making process [14]. So, based on the enrichment potential demonstrated by *Limma* and ML it is feasible to expect a significant confidence gain by incorporating these two independent approaches. From Table 4 can be confirmed the relevance of PD_02 and PD_07 for PD from a ML and/or *Limma* perspective. Here, we use an intuitive measure of the merit of each module based on the number of genes in the module identiffied by each approach. The merit values of ML and/or *Limma* associated to PD_02 and PD_07 outperform from 1.3-fold to 3.8-fold the closest module (PD_04).

***Statistical Significance****.* In order to statistically validate our module prioritization strategy each WGCN PD module was subject to a hypergeometric probability test. Detailed results are provided in Table 6. From this table it is possible to note that only PD_02 is enriched in PD related genes significantly beyond what might be expected by chance (p-value = 0.0034) while PD_07 is in the limits of the statistical significance (*p*-value = 0.0512). These results support the strategy followed for modules prioritization. Regarding to the inclusion of the module PD_07, as previously mentioned, the GAD database was used just as a common reference framework for comparison purposes. Therefore, the p-values reported must be used as a decision-making criterion instead of a definitive selection/rejection criterion. On the other hand, the biological relevance of this module also grants its inclusion as will be demonstrated in the following section.

**Table 6:** Hypergeometric test results for the WGCN PD modules based on 319 known PD related genes in GAD and 8477 background genes

Prioritized PD Module	*n*	*m*	*p*-value
PD_01	603	29	0.1014
PD_02	1437	73	0.0034
PD_03	133	6	0.3849
PD_04	161	6	0.5685
PD_05	789	19	0.9897
PD_06	468	15	0.7776
PD_07	494	26	0.0512
PD_08	213	10	0.2813
PD_09	4179	128	0.9997
PD_02 ∪ PD_07	1931	99	0.0003

*n*: number or genes in the prioritized PD module; *m*: number of known PD related genes in GAD found in the prioritized module; *p*-value: hypergeometric probability of finding by chance *k* or more known PD related genes in a set of *n* prioritized genes

**Biological Relevance**. The space of biological process covered by the respective PD_02 and PD_07 gene sets was explored by conducting a joined gene ontology (GO) enrichment analysis in order identify commonalities and uniqueness between these two modules. The association between the corresponding biological process and PD were contrasted with the current literature evidence. The full details on the enrichment analysis are provided as supplementary information (see Additional file 5).

From this analysis four processes well known to be associated with PD can be highlighted from the 1437 genes included in the module PD_02: oxidative phosphorylation; intracellular transport; mitochondrion organization; and learning or memory. These results reflect the well-known mitochondrial complex I deficiency [59] (specifically, primary defects in mitochondrial oxidative phosphorylation [60]) leading to oxidative stress, largely associated to PD and their characteristics motor and cognitive impairments [59–63]. In terms of biological processes, the information provided by the genes included in this module and those prioritized by *Limma* is highly consistent. Even so, contrary to *Limma* prioritization, this module do not enrich mainly dopamine metabolism processes but also energetic process. This suggest that the dopamine bias could be actually compensated by combining *Limma* and co-expression analysis.

From the 494 genes involved in PD_07 three processes well known to be associated with PD can be highlighted: protein folding; response to unfolded protein; and response to protein. These processes had being largely reported by other authors [48, 49, 53] and could be associated with the role of α-Synuclein misfolding and aggregation in the pathogenesis of PD [64].

**Fig. 2:**
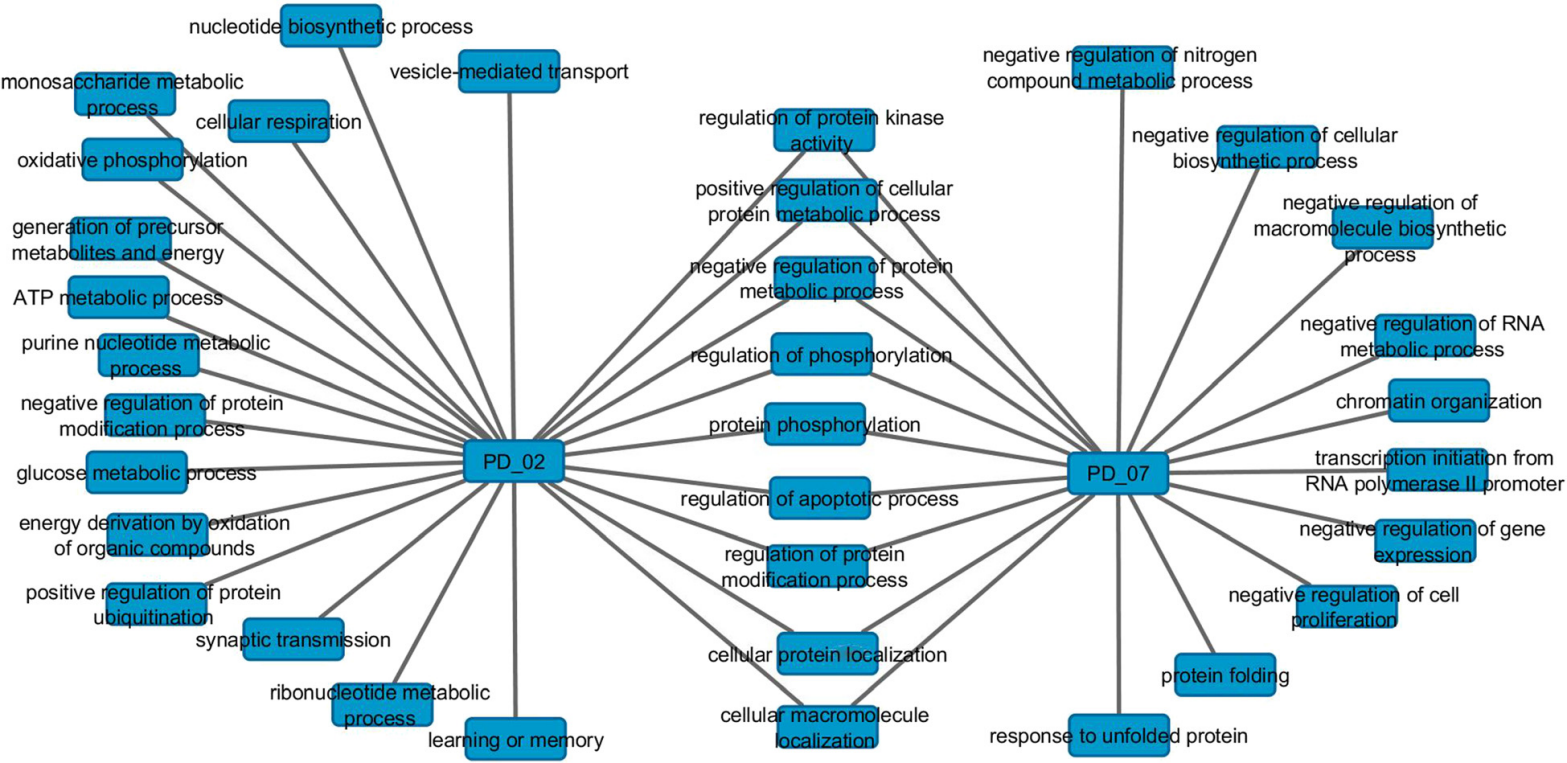
Representative common and unique biological process covered by modules PD_02 and PD_07

A combined enrichment analysis of the biological process comprised in PD_02 and PD_07 was conducted with aid of the ToppCluster tool [41] (see details in the Additional file 5). The resultant network representation of individual and common biological process for PD_02 and PD_07 is provided in Fig. 2. As can be noted in this figure, both modules share common biological processes including the influence in protein phosphorylation, apoptosis and protein metabolism. Some of these processes, such as oxidative phosphorylation and apoptosis has been extensively reported in PD [46–49, 51, 53], while other process mainly related with post-translational and post-transcriptional modifications have been less explored in PD [48, 49].

For example, SNCA is present in PD_02, however, most of the histones and chaperones are located in PD_07. Specifically the heat shock protein family B (small) member 1 (HSPB1) is included in PD_07. This gene has long been associated with PD [53, 65]. In addition to protein folding this gene is also involved in the apoptosis pathway (11) which is common to both modules. While PD_02 mainly covers energetic and synaptic biological process (oxidative fosforilation, energy metabolism, synaptic transmision and memory), PD_07 is more focused in processes related with folding and transcription regulation origins (protein folding; response to unfolded protein; and response to protein). By considering both modules we are covering not only common biological processes relevant for PD but also other process equally relevant for PD but uniquely covered by the respective module. So, PD_07 not only covers biological process significantly related to PD but also includes some biological process equally significant for PD which are not covered by PD_02.

### Consensus gene prioritization strategy

The results obtained in WGCN modules prioritization suggest that the consensus use of several independent sources of information significantly contribute to identify genes sets statistically and biologically relevant to PD. In doing so, all the independent prioritization analyses made (*Limma*, ML, and WGCN analyses) were combined in a consensus gene prioritization strategy. Finding a consensus based on all these tools can provide reliable, statistically significant and biologically relevant genes sets highly enriched with already known and potentially novel PD related genes [14]. The proposed consensus strategy is really simple, but also highly effective as will be demonstrated:

*Only those genes jointly identified by ML and Limma analysis (common genes) and also present in the biologically relevant WGCN modules PD_02 or PD_07 can be considered as statistically and biologically relevant for PD.*

This consensus strategy based in the common interception of three conceptually different prioritization strategies is actually a highly stringent approach. However; such stringent criteria should provide a desirable balance of enrichment and biological significance of the prioritized gene list.

Our strategy provides a genes list sorted in decreasing order of probability of association with PD by applying fusion rules (*Min-* and *Mean-Rank*) based on *Limma* and ML ranks. That is, genes are first sorted according to the minimum rank assigned by ML and *Limma*, and then by the average of ML and *Limma* ranks.

Following the proposed consensus strategy was prioritized a set of 50 genes sorted in a decreasing order of relevance for PD. Details on this genes set are provided in Additional file 1: Table S3. As can be noted in the table, 7 out 50 (TP rate = 14 %) genes were found in the set of 319 known PD related genes in GAD. However, after an exhaustive literature search for associations between each of the 50 genes and PD was possible to establish direct associations for 20 genes in this prioritized set (TP rate = 40 %).

***Statistical Significance****.* The statistical validity of the consensus strategy needs to be challenged and compared with the rest of the alternative gene prioritization options. For this, the hypergeometric test, and the random bootstrap sampling were applied to the genes set prioritized by the consensus strategy, the ML and *Limma* analysis (independently and in combination) as well as to the genes set corresponding to PD_02 and PD_07 (independently and in combination). See details in Table **7**.

**Table 7:** Statistical validation of the different gene prioritization strategies employed in this work (independently and in combination). Hypergeometric test, random bootstrap sampling experiment and enrichment features of the different gene prioritization strategies

	Hypergeometric Test			Random Bootstrap Sampling (100 Generations)						Enrichment	
	*n*	*m*	*p-value*	*Mean*	*Median*	*Min.*	*Max.*	*Std. Dev.*	*p-value (W)*	*Fold-Enrichment*	*TP Rate*
*Limma*	134	10	0.0295	5.0410	5	0	17	2.1852	<0.0001	1.9837	0.0746
ML	168	11	0.0520	6.3211	6	0	22	2.4421	<0.0001	1.7402	0.0655
ML ∪ *Limma*	246	14	0.0805	9.2609	9	0	25	2.9426	<0.0001	1.5117	0.0569
PD_02	1437	73	0.0034	55.4259	55	25	87	6.6392	<0.0001	1.3171	0.0508
PD_07	494	26	0.0512	18.5957	18	2	41	4.1038	<0.0001	1.3982	0.0526
PD_02 ∪ PD_07	1931	99	0.0003	72.6709	73	37	112	7.3516	<0.0001	1.3623	0.0513
Concensus	50	7	0.0025	1.8817	2	0	10	1.3407	<0.0001	3.7200	0.1400

*n*: number or genes in the prioritized PD module; *m*: number of known PD related genes in GAD found in the prioritized module; *p-value*: hypergeometric probability of finding by chance *k* or more known PD related genes in a set of *n* prioritized genes; *Mean*/*Median*/*Min.*/*Max.*/*Std. Dev.*: average/median/minimum/maximum/standard deviation of the number of known PD related genes in GAD included in randomly selected gene sets with the same number of genes as the corresponding set of prioritized genes; *Fold-enrichment*: fold difference between *m* and *Mean* (*Fold-enrichment = m*/*Mean*); *TP Rate*: ratio of known PD related genes in *n* (*TP Rate = m*/*n*)

As deduced from the hypergeometric test, not every genes set prioritized can be considered as statistically significant. Although “PD_02 ∪ PD_07” looks like the better option, its significantly higher number of genes compared with “Consensus” hinders its potential for prioritization tasks. Actually, the TP rate of the “Consensus” strategy with only 50 genes is almost three-folds.

Based on the random bootstrap sampling experiment no genes set seems to be randomly enriched with known PD related genes. Again, the consensus strategy stands out for a significantly higher enrichment with known PD related genes compared with the corresponding random enrichment determined in the experiment (*Fold-Enrichment*). The consensus strategy is about four times more enriched in known PD related genes than might be expected by chance, which is almost two-fold compared with “*Limma*”, the nearest strategy according to *Fold-Enrichment*.

*Enrichment and Early Recognition Ability.* Due to the high cost associated to the experimental validation of gene-disease associations and the high number of candidate genes initially considered (thousands), the early recognition ability of a gene prioritization tool should be considered as the ultimate measure of its utility [16]. The estimation of the early recognition ability by statiscally sound metrics is well established in chemoinformatics as part of the validation of virtual screening tools. In this work we propose, for the first time, the use of such metrics for gene prioritization tasks.

From the accumulation curve we can deduce overall enrichment from the area under this curve (*AUAC*) which is defined as:

3 $$ AUAC = 1-\frac{1}{n}{\displaystyle {\sum}_{i=1}^n}{x}_i $$

where *n* is the total number of known disease-related genes in the total background gene set (*N*) and *x*_*i*_ is the relative rank of the *i-*th known disease-related gene in the ordered list when their corresponding rank *r*_*i*_ is scaled to *N*, (*x*_*i*_ = *r*_*i*_*/N*). So, *AUAC* can be interpreted as the probability that a known disease-related gene, selected from the empirical cumulative distribution function defined by the rank-ordered list, will be ranked before a gene randomly selected from a uniform distribution [17].

The (Receiver Operating Characteristic) ROC curve describes the true positives rate (*TP rate*) for any possible change of the number of selected genes as a function of the false positives rate (*FP rate*) [66]. The area under the ROC curve (*ROC*) can be interpreted as the probability that a known disease-related gene will be ranked earlier than a disease-unrelated gene within a rank-ordered list [17]. The *ROC* metric is defined as:

4 $$ ROC=\frac{AUAC}{R_i}-\frac{R_a}{2{R}_i} $$

where *R*_*a*_ = *n*/*N*, and stands for the ratio of known disease-related genes in the dataset, whereas *R*_*i*_ = *N*-*n*/*N*, and represents the ratio of disease-unrelated genes in the total background gene list.

On the other hand, the enrichment factor (*EF*) takes into account the improvement of the hit rate by a gene prioritization protocol compared to a random selection. This metric has the advantage of answering the question: how enriched in known disease-related genes, the set of *n* genes that I prioritize will be, compared to the situation where I would just pick the *n* genes randomly?

5 $$ EF = \frac{\raisebox{1ex}{$m$}\!\left/ \!\raisebox{-1ex}{$n$}\right.}{\raisebox{1ex}{$M$}\!\left/ \!\raisebox{-1ex}{$N$}\right.} $$

where *n* is the number of genes in the filtered fraction (χ) and *m* is the number of known disease-related genes retrieved at this fraction, being χ determined by the quotient between *n* and *N* (χ = *n*/*N*). The maximum value that *EF* can take is 1/χ if χ ≥ *M*/*N, N*/*M* if χ < *M*/*N*, and the minimum value is zero [17].

However, the “early recognition” ability of a prioritization tool is encoded by just a few enrichment metrics such as the robust initial enhancement (*RIE*) and the Boltzmann-enhanced discrimination of ROC (*BEDROC*) metrics [17]. The *RIE* metric describes how many times the distribution of the ranks for known disease-related genes caused by a prioritization protocol is better than a random rank distribution and is defined as:

6 $$ RIE = \frac{{\displaystyle {\sum}_{i=1}^n}{e}^{-\alpha {x}_i}}{\frac{M}{N}\left(\frac{1-{e}^{\alpha }}{e^{\raisebox{1ex}{$\alpha $}\!\left/ \!\raisebox{-1ex}{$N$}\right.}-1}\right)} $$

The parameter α is used to assign a higher weight (and so a higher contribution to the *RIE* metric) to known disease-related genes ranked at the beginning than those at the end of the ordered list and can be interpreted as the fraction of the list where the weight is important. Specifically, in this work the *RIE* and also *EF* and *BEDROC* metrics were evaluated at χ = 1 %/5 %/10 %/20 %, which corresponds to values of α = 160.9/32.2/16.1/8, respectively.

However, like *EF*, *RIE* depends on *N*, *R*_*a*_ and α, which hampers its use in datasets of different size and composition. The other limitation is that unlike *ROC*, *RIE* neither provides a probabilistic interpretation nor a measurement of the enrichment performance above all thresholds [66].

In order to derive a new metric overcoming these limitations Truchon and Bayly proposed the *BEDROC* metric [17].

7 $$ BEDROC = \frac{RIE-RI{E}_{min}}{RI{E}_{max}-RI{E}_{min}} $$

*RIE*_*min*_ and *RIE*_*max*_ are obtained when all the known disease-related genes are at the beginning and at the end of the ordered list, respectively.

8 $$ RI{E}_{min} = \frac{1-{e}^{\alpha {R}_a}}{R_a\left(1-{e}^{\alpha}\right)} $$

9 $$ RI{E}_{max} = \frac{1-{e}^{-\alpha {R}_a}}{R_a\left(1-{e}^{-\alpha}\right)} $$

The *BEDROC* metric is a generalization of the *ROC* metric that includes a decreasing exponential weighting function that adapts it for use in early recognition problems. This metric can be interpreted as the probability that a known disease-related gene ranked by a prioritization protocol will be found before a gene that would come from a hypothetical exponential probability distribution function with parameter α. Thus, *BEDROC* should be understood as a “prioritization usefulness scale” [17].

From the seven prioritization strategies being compared, in Table 8 we estimate and compare the respective overall enrichment and early recognition ability of those four providing a ranked list of genes through all or part of the initial background of 8477 candidate genes.

**Table 8:** Overall enrichment and early recognition metrics of the four prioritization strategies considered

	*Limma*	*ML*	*ML-Limma*	*Consensus*
Classic Enrichment Metrics				
*AUAC*	0.498	0.502	0.495	0.540
*ROC*	0.498	0.502	0.495	0.541
*EF*_*1%*_	2.855	2.521	2.847	3.164
*EF*_*5%*_	1.449	1.387	1.007	1.512
*EF*_*10%*_	1.038	1.385	0.913	1.510
*EF*_*20%*_	0.975	1.054	1.054	1.321
Early Recognition Metrics				
*RIE*_*1%*_	2.452	2.213	2.403	2.577
*RIE*_*5%*_	1.286	1.438	1.157	1.583
*RIE*_*10%*_	1.089	1.225	1.044	1.400
*RIE*_*20%*_	1.021	1.085	1.008	1.230
*BEDROC*_*1%*_	0.094	0.086	0.094	0.099
*BEDROC*_*5%*_	0.091	0.102	0.083	0.113
*BEDROC*_*10%*_	0.131	0.147	0.125	0.168
*BEDROC*_*20%*_	0.216	0.230	0.214	0.262

The ranking provided through the full list of 8477 genes by each strategy is defined by the respective scoring factor employed in the gene prioritization process. Since just a subset of genes is prioritized by each strategy, only this fraction is ranked and the remaining genes in the full list of 8477 genes are randomized. The rationale of such a experiment design is to resemble as much as possible the respective prioritization strategy. This randomization strategy is prefered over just to evaluate the respective metrics on the respective prioritized genes set in order to avoid the saturation effect present in small sets with a high ratio of known disease-related genes [17]. The goal here is to evaluate the ability of each prioritization strategy to retrieve the highest fraction possible of those 319 known PD relevant genes in the earliest possible fraction of the respective ordered list. The exact composition of the four respective lists (including ranking and aleatorization rules) is detailed in the supplementary information.

All the values corresponding to *AUAC* and *ROC* metrics provided in Table 8 are close to 0.5, reflecting that the overall enrichment ability of the four prioritization strategies is not better than a random selection. This result, although expected due to the fact that >90 % of the candidate genes are randomized must not be interpreted as a lack of utility of the prioritization strategies. Instead, the real estimation of their utility must focuse on their early recognition ability.

**Fig. 3:**
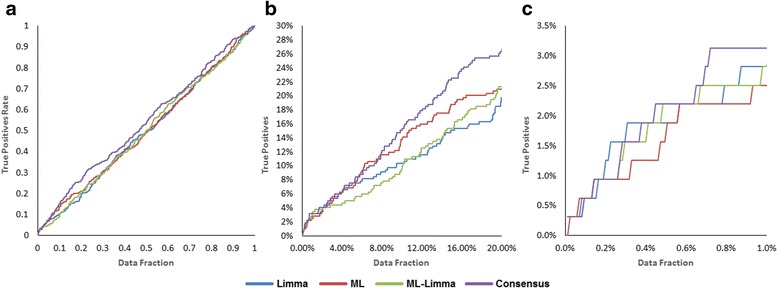
Accumulation curves of the four prioritization strategies considered. Overall enrichment represented by the accumulation curve for the full set of 8477 background genes for the respective prioritization strategies (**a**). Zoom of the top 20 %/1 % fraction of the ordered list providing information on the early recognition ability of the respective prioritization strategies (**b**/**c**)

The corresponding values of *EF* at the top fractions studied (1, 5, 10, and 20 %) as well as the early recognition metrics (*RIE*, and *BEDROC*) show that the Consensus strategy compares favorably over the rest of strategies considered, but the difference looks minimal. However, the use of biologically relevant information from PD_02 and PD_07 highlights the advantages of using the Consensus strategy. The comparative overall enrichment and early recognition performance of the four prioritization strategies can be visually confirmed on Fig. 3. As can be noted in Fig. 3b, the enrichment performance of the Consensus strategy clearly outperforms the other three strategies on the top 20 % fraction of the list of 8477 genes considered. The same trend is confirmed in the top 1 % fraction (see Fig. 3c), the most relevant fraction to consider for early recognition assessment [16].

Finally, we evaluated whether each of these prioritization methods ranks a set of known PD genes significantly early than an alternative method. For this, we applied a Wilcoxon signed rank test to compare the ranking provided by the four approaches under study (*Limma*, ML, ML-*Limma* and Consensus) for the 100 % and the top 20 %/10 %/5 % of the 319 PD genes collected from GAD. From this analysis is possible to note that although there is not an evident difference between the early recognition metrics of the four approaches, the consensus strategy ranks the PD genes significantly early than the other three approaches (*Limma*, ML and ML- *Limma*) in all the fractions analyzed [100 % (319 PD Genes in GAD), top 20 % (top 64 PD genes), top 10 % (top 32 PD genes) and top 5 % (top 16 PD genes)]. Only the ranking provided by the consensus strategy for the top 16 PD genes (top 5 %) was not significantly better than the ranking provided by *Limma*. See Table 9 for details.

**Table 9:** Results of the Wilcoxon signed rank test conducted to compare the ranking provided by the four approaches under study

319 PD Genes in GAD (100 %)				
	Limma	ML	ML-Limma	Consensus
Limma	(−−−)	2.62E-09	2.85E-01	2.79E-62
ML	2.62E-09	(−−−)	1.16E-01	4.36E-47
ML-Limma	2.85E-01	1.16E-01	(−−−)	4.27E-64
Consensus	2.79E-62	4.36E-47	4.27E-64	(−−−)
64 Top Ranked PD Genes in GAD (Top 20 %)				
	Limma	ML	ML-Limma	Consensus
Limma	(−−−)	1.84E-05	6.09E-01	6.81E-09
ML	1.84E-05	(−−−)	1.21E-07	1.69E-06
ML-Limma	6.09E-01	1.21E-07	(−−−)	1.24E-12
Consensus	6.81E-09	1.69E-06	1.24E-12	(−−−)
32 Top Ranked PD Genes in GAD (Top 10 %)				
	Limma	ML	ML-Limma	Consensus
Limma	(−−−)	7.19E-01	5.23E-04	1.19E-02
ML	7.19E-01	(−−−)	7.25E-02	3.11E-02
ML-Limma	5.23E-04	7.25E-02	(−−−)	1.38E-05
Consensus	1.19E-02	3.11E-02	1.38E-05	(−−−)
16 Top Ranked PD Genes in GAD (Top 5 %)				
	Limma	ML	ML-Limma	Consensus
Limma	(−−−)	6.06E-01	4.23E-01	3.02E-01
ML	6.06E-01	(−−−)	3.02E-01	1.95E-03
ML-Limma	4.23E-01	3.02E-01	(−−−)	4.33E-02
Consensus	3.02E-01	1.95E-03	4.33E-02	(−−−)

***Biological Relevance****.* Since the final 50 genes comes from the intersection of the prioritizations made by *Limma*, WGCNA modules, and specially ML, a reduced statistical significance of their biological processes should be expected too, similarly to ML. Most of the top enriched GO terms in the biological process enrichment analysis are associated with PD: dopamine (DA) metabolism [59–63, 67–80]; prepulse inhibition (PPI) [81–86]; metal ion transport and pigmentation [87–96]. None of the biological processes is statistically significant by using an FDR adjusted p-value < 0.05 as significance cutoff. See details in the supplementary information. However, from the top ten GO terms only one is directly related with dopamine metabolism pointing to a reduced dopamine bias.

Additionally, an exhaustive literature search was conducted in order to find direct or indirect evidence of the association with PD of each of the 50 genes prioritized. As “direct evidence” we considered scientific publications reporting a relationship (i.e. mutation, expression or knockout) between the gene and PD. As “indirect evidence” we considered scientific publications reporting a theoretical (i.e. system biology) or experimental (i.e. mutation, expression, knockout) evidence of the association of the gene with already known targets or biological processes known to be related with PD pathogenesis.

The microarrays used in our study as raw data correspond to references [45–47, 53, 97]. No result coming from these studies only was used as “evidence”. However, studies performing system biology analysis which include also our microarrays were considered because the strategy for data exploration was different and therefore we don’t necessarily have to agree in the establishment of genes-diseases association. However, even those studies were considered as “indirect evidence”. Any studies carried on in different microarrays and reporting a down/up regulation were considered also but as “indirect evidence”.

The literature review conducted evidenced that 20 out of the 50 candidate genes were directly associated with PD (SLC18A2; AGTR1; GBE1; PDCD2; ALDH1A1; SLC6A3; TH; HIST1H2BD; DRD2; EN1; TRIM36; FABP7; PTPRN2; VWA5A; ITPR1; CACNB3; CHORDC1; NDUFA9; RGS4; SNRNP70). Additionally, indirect evidence of association with PD was found for another 8 genes (CCNH; DLK1; PCDH8; SLIT1; BMI1; DLD; PBX1; INSM), which are potentially new therapeutic targets or biomarkers for PD. Details on the direct or indirect literature evidence supporting the association with PD of many of the 50 genes prioritized by our consensus strategy are provided in Table 10.

**Table 10:** Literature evidence of the association with PD for the 50 genes prioritized with the consensus strategy

Official Gene symbol	Direct Evidence	Indirect Evidence	Description
SLC18A2	1	0	Several studies reported the association between SLC18A2 and PD [117–121]. In humans, the involvement of SLC18A2 in PD pathogenesis is supported by positron emission tomography studies showing significantly lower SLC18A2 densities in the putamen, caudate, and SN of PD patients [122–125]. Its potential as PD biomarker [118] or even as a PD pharmacological target [126] have also been suggested. A method of diagnosing PD comprising a set of differentially expressed genes including SLC18A2 was patented [127].
AGTR1	1	0	AGTR1 have been significantly and consistently downregulated in several PD microarray studies [46, 47, 53, 128, 129]. Additionally, the protective effects on dopaminergic neurons of AGTR1 inhibitors have been well documented [130–136] highlighting the role of AGTR1 as a potential pharmacological target in PD.
GBE1	1	0	GBE1 has been found to be downregulated in gene expression profiling studies of human *substantia nigra pars compacta* from PD patients employing high density microarrays [121, 137]. A method of diagnosing PD comprising a set of differentially expressed genes including GBE1 was patented [127].
PDCD2	1	0	The isoform 1 of PDCD2 was found to be ubiquitinated by parkin and increased in the *substantia nigra* of patients with both autosomal recessive and sporadic PD [138].
ALDH1A1	1	0	ALDH1A1 has been found to be significantly and consistently downregulated in several PD microarray studies [46, 47, 53, 121, 128, 129, 137, 139] highlighting DA metabolism dysfunction resulting in oxidative stress and most probably leading to neuronal cell death. Two methods of diagnosing PD comprising a set of differentially expressed genes including ALDH1A1 were patented [127, 140].
CCNH	0	1	So far, cyclin H (CCNH) has not been directly linked to the pathogenesis of PD. However, the cyclin-dependent kinase 5 (CDK5) was found to act as a mediator of dopaminergic neuron loss in a mouse model of Parkinson’s disease [141], pointing the potential role of CCNH as a novel and unexplored PD biomarker.
NRXN3	0	0	No association between NRXN3 and PD was found.
SLC6A3	1	0	A combined analysis of published case–control genetic associations between SLC6A3 and PD involving several ethnicities provided evidences of the role of SLC6A3 as a modest but significant risk factor for PD [142].
DLK1	0	1	No direct associations between DLK1 and PD have been reported. However, through a combined gene expression microarray study in NURR1(−/−) mice DLK1 was identified as novel NURR1 target gene in meso-diencephalic DA neurons [143]. NURR1 (also known as NR4A2) encodes a member of the steroid-thyroid hormone-retinoid receptor superfamily [144]. Mutations in this gene have been associated with disorders related to dopaminergic dysfunction including PD [145–163].
GPR161	0	0	No association between GPR161 and PD was found.
SCN3B	0	0	No association between SCN3B and PD was found.
TH	1	0	TH has been largely associated with PD [164–167].
PCDH8	0	1	No direct association between PCDH8 and PD was found unless a network-based systems biology study utilizing several PD-related microarray gene expression datasets and biomolecular networks [168].
ORC5	0	0	No association between ORC5 and PD was found.
HECA	0	0	No association between HECA and PD was found.
SLIT1	0	1	No direct association between SLIT1 and PD was found. However, the axonal growth inhibition of fetal and embryonic stem cell-derived dopaminergic neurons reported for SLIT1 [169] suggest an indirect association with PD.
BMI1	0	1	Although BMI1 has not been directly associated with PD a previous study demonstrated that it is required in neurons to suppress apoptosis and the induction of a premature aging-like program characterized by reduced antioxidant defenses [170]. These findings provide a molecular mechanism explaining how BMI1 regulates free radical concentrations and reveal the biological impact of BMI1 deficiency on neuronal survival and aging. The activity of BMI1 against mitochondrial ROS may be also relevant to age-associated neurodegenerative diseases where cell death is apparently mediated by oxidative damage, such as in Parkinson disease [171].
QPCT	0	0	No association between QPCT and PD was found.
DLD	0	1	No direct association between DLD and PD was found. However, mice that are deficient in DLD [172] exhibited an increased vulnerability to 1-methyl-4-phenyl-1,2,3,6-tetrahydropyridine (MPTP) [173], which have been proposed for use in models of PD [174]. DLD is a critical subunit of key mitochondrial enzyme complexes such as the ketoglutarate dehydrogenase complex (KGDHC) and the pyruvate dehydrogenase complex (PDHC) [175]. Altered energy metabolism, including reductions in KGDHC and PDHC are characteristic of many neurodegenerative disorders including PD [176, 177].
HIST1H2BD	1	0	HIST1H2BD was found to be significantly and differentially expressed in 20 out of the 21 brain regions studied in a multiregional gene expression analysis in postmortem brain coming from 23 control and 22 PD cases [178]. A method of diagnosing PD comprising a set of differentially expressed genes including HIST1H2BD was patented [179].
PBX1	0	1	No direct association between PBX1 and PD was found. However, the expression of PBX1 in dopaminergic neurons make it an important player in defining the axonal guidance of the midbrain dopaminergic neurons, with possible implications for the normal physiology of the nigro-striatal system as well as processes related to the degeneration of neurons during the course of PD [180].
SRP72	0	0	No association between SRP72 and PD was found.
DRD2	1	0	DRD2 has been largely associated with PD [181–194].
EN1	1	0	Several studies have reported significant associations between EN1 and PD [195–197].
TRIM36	1	0	TRIM36 has been found to be downregulated in a gene expression profiling study of human *substantia nigra pars compacta* from PD patients employing high density microarrays [137]. A method of diagnosing PD comprising a set of differentially expressed genes including TRIM36 was patented [127].
INSM1	0	1	Although INSM1 has not been directly associated with PD a previous study demonstrated that it is involved on the interrelation of odor and motor changes probably caused by a Mn-induced dopaminergic dysregulation affecting both functions [198]. In this study was found that the rs2871776 G allele, which was associated with the worst effect of Mn on motor coordination, was linked to alteration of a binding site for the transcription factor INSM1. This gene plays an important role in the developing CNS, and especially of olfactory progenitors, as shown in mouse [199] and human [200] embryos. Olfactory impairment is a highly recurrent non-motor dysfunction in PD and is considered an early predictive sign of neurodegeneration [201–203].
MDH2	0	0	No association between MDH2 and PD was found.
CIRBP	0	0	No association between CIRBP and PD was found.
FABP7	1	0	A recent study reported that FABP7 levels were elevated in serum of 35 % of the patients with PD and only in 2 % of the healthy controls, suggesting the role of FABP7 as a potential biomarker for PD [204]. FABP7 was also identified as a promising candidate in a previous quantitative trait loci (QTL) study conducted to identify genes that mediate PPI in mice [205]. This finding was confirmed in a further experiment where FABP7-deficient mice showed decreased PPI. PPI deficiencies is considered a characteristic indicator of schizophrenia [82], but is also deficient in PD patients [206, 207].
PTPRN2	1	0	PTPRN2 has been found to be downregulated in a gene expression profiling study of human *substantia nigra pars compacta* from PD patients employing high density microarrays [137]. A method of diagnosing PD comprising a set of differentially expressed genes including PTPRN2 was patented [127].
PSMG1	0	0	No association between PSMG1 and PD was found.
VWA5A	1	0	VWA5A was associated with PD through a genome-wide genotyping study in PD and neurologically normal controls [208].
ITPR1	1	0	Kitamura et al. [209] reported since 1989 that ITPR1 binding sites were reduced by about 50 % in several brain regions of PD patients (caudate nucleus, putamen, and pallidum) as compared to findings in the age-matched controls, suggesting a probable implication of ITPR1 in PD.
BAI3	0	0	No association between BAI3 and PD was found.
CPT1B	0	0	No association between CPT1B and PD was found.
CACNB3	1	0	The calcium channel subunit b3 (CACNB3), the ATPase type 13A2 (PARK9), and several subunits of Ca^2+^ transporting ATPases (ATP2A3, ATP2B2, and ATP2C1) were downregulated in PD further substantiating the involvement of a deficit in organelle function and of Ca^2+^ sequestering.
ACP2	0	0	No association between ACP2 and PD was found.
CHORDC1	1	0	CHORDC1 was found to be significantly and differentially expressed in 19 out of the 21 brain regions studied in a multiregional gene expression analysis in postmortem brain coming from 23 control and 22 PD cases [178]. A method of diagnosing PD comprising a set of differentially expressed genes including CHORDC1 was patented [179].
SHOC2	0	0	No association between SHOC2 and PD was found.
VBP1	0	0	No association between VBP1 and PD was found.
PPM1B	0	0	No association between PPM1B and PD was found.
YME1L1	0	0	No association between YME1L1 and PD was found.
NDUFA9	1	0	NDUFA9 is included in the KEGG Parkinson’s Disease Pathway (http://www.genome.jp/dbget-bin/www_bget?pathway+hsa05012).
TRAPPC2L	0	0	No association between TRAPPC2L and PD was found.
HIST1H2AC	0	0	No association between HIST1H2AC and PD was found.
RGS4	1	0	RGS4 was found to be significantly and differentially expressed in several brain areas of postmortem samples coming from PD patients in comparison to control samples [53]. On the other hand, experiments in mice with reserpine-induced acute DA depletion suggest that RGS4-dependent attenuation of interneuronal autoreceptor signaling is a major factor in the elevation of striatal acetylcholine release in PD [210]. Lerner and Kreitzer [211] also identified RGS4 as a key link between DA 2/adenosine 2A signaling and endocannabinoid mobilization pathways. In addition, in contrast to wild-type mice, RGS4 deficient mice exhibited normal endocannabinoid-dependent long-term depression after DA depletion and were significantly less impaired in the 6-OHDA model of PD. Taken together, these results suggest that inhibition of RGS4 may be an effective nondopaminergic strategy for treating Parkinson’s disease. Finally, RGS4 was recently found to be involved in the generation of abnormal involuntary movements in the unilateral 6-hydroxydopamine (6-OHDA)-lesioned rat model of PD [212].
CRYZL1	0	0	No association between CRYZL1 and PD was found.
RCN2	0	0	No association between RCN2 and PD was found.
SNRNP70	1	0	SNRNP70 was associated with woman affected by PD in an association study of four common polymorphisms in the DJ1 gene and PD involving 416 PD probands and their unaffected siblings matched by gender and closest age [213].
VPS4B	0	0	No association between VPS4B and PD was found.

As previously mentioned, the most relevant feature of the consensus gene prioritization strategy proposed is the early recognition ability evidenced [17]. It is significant that the first 5 genes prioritized (first 10 %) could be confirmed with direct literature evidence. Finally, it is worthy to note that based on the hypergeometric test it is possible to assert that the identification of 20 or more genes out of up to 2402 known PD related genes in a set of 50 prioritized genes is still significantly distant from being a random selection (*p*-value = 0.049867). That is, considering that an additional set of genes apart of those currently reported in GAD can be relevant for PD but unreported up today, the prioritized list of 50 genes is still statistically significant even in the case that the actual (unknown) set of PD relevant genes would be more than 7-fold (2402) those currently reported in GAD (319).

Considering the above mentioned in addition to the reduced size of the final set of genes prioritized by the consensus strategy we conducted an additional analysis. This analysis was based on the construction of a functional interaction network with the aid of the *Search Tool for the Retrieval of Interacting Genes/Proteins* (STRING) [98, 99] from this final set of 50 genes prioritized with the consensus strategy (actually less because some of these genes don’t have reported interaction in our space) and 100 additional interacting genes with a confidence score higher than 0.7. This network was imported into Cytoscape [100] and each gene node was labeled in order to differentiate those genes in the 50 genes prioritized with the consensus strategy from the 100 additional interacting genes added with STRING. The resultant network representation in provided in the supplementary information (see Additional file 5).

**Fig. 4:**
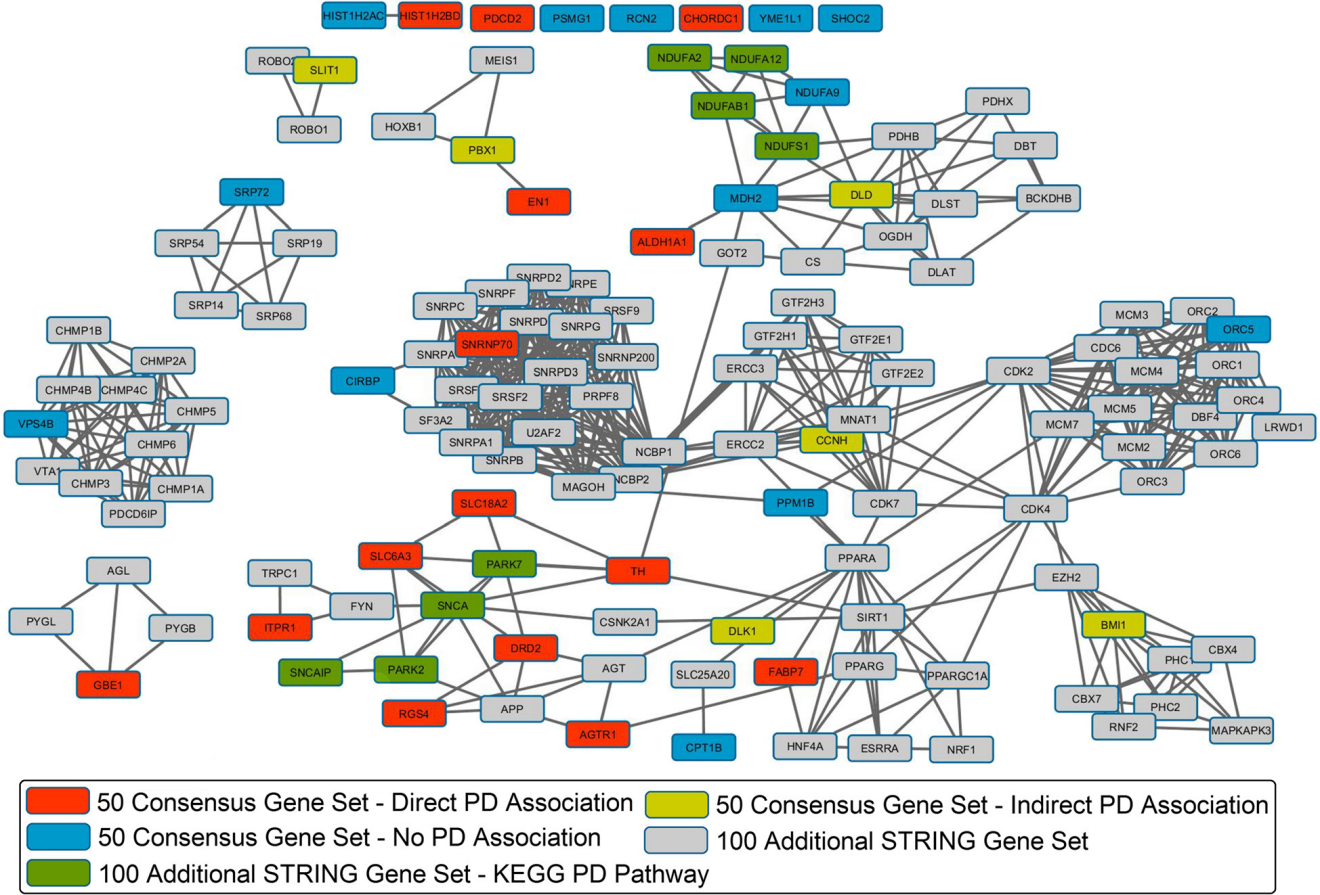
Functional interaction network of the final set of 50 genes prioritized with the consensus strategy and 100 additional interacting genes. Each gene node was labeled in order to differentiate those genes in the 50 genes prioritized with the consensus strategy from the 100 additional interacting genes (labeled in *gray*). Genes with direct, indirect and no literature evidences of association with PD among the 50 genes prioritized with the consensus strategy were labeled in *red*, *yellow* and *blue*, respectively. Those genes among the 100 additional interacting genes included in the KEGG PD pathway were labeled in *green*

This network includes ubiquitin C (UBC), which appears as a central gene connecting most of the genes included in the network. Although the role of UBC and related genes/proteins in PD through biological process such as protein synthesis, folding and degradation has long been established [52, 101, 102], their hub nature in our network could induce a connectivity bias at the time to perform further visual interaction or biological processes enrichment analysis. So UBC was removed from the network previous to conduct the mentioned analysis. Details on the biological processes enrichment analysis are provided in the supplementary information. The functional interaction network after removing UBC is provided in Fig. 4.

**Fig. 5:**
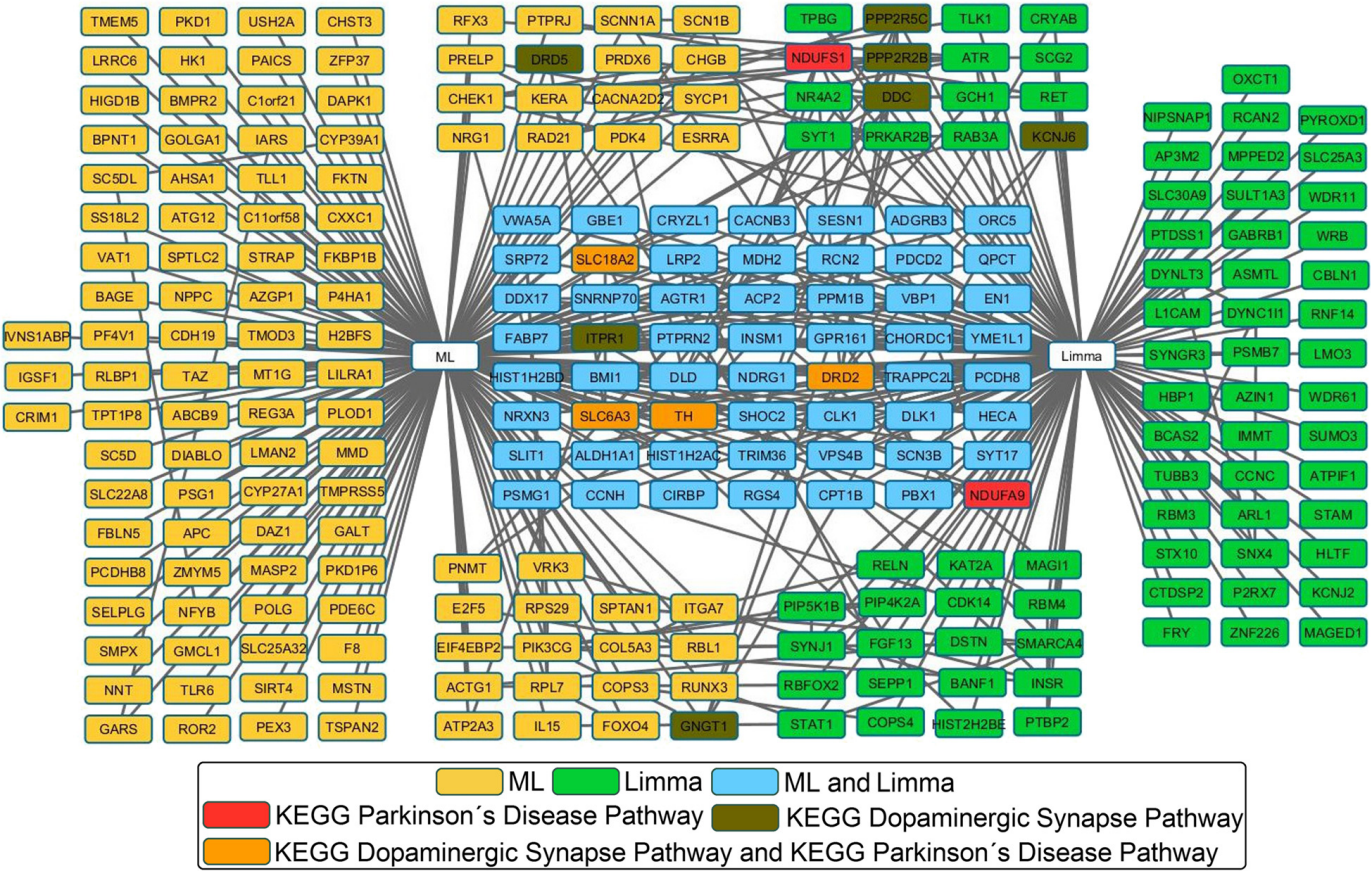
Functional interaction network comprising gene sets prioritized by *Limma* and ML, respectively. The genes prioritized by ML/*Limma* only are represented by *yellow/green* nodes, while those genes prioritized by both approaches (ML and *Limma*) are represented by *blue* nodes. Genes in the KEGG Dopaminergic Synapse Pathway/KEGG Parkinson’s Disease Pathway are represented by *olive/red* nodes, while those genes included in both pathways (Dopaminergic Synapse and Parkinson’s Disease) are represented by *orange* nodes

From the literature search 22 genes (NRXN3, GPR161, SCN3B, ORC5, HECA, QPCT, SRP72, MDH2, CIRBP, PSMG1, BAI3, CPT1B, ACP2, SHOC2, VBP1, PPM1B, YME1L1, TRAPPC2L, HIST1H2AC, CRYZL1, RCN2, VPS4B) were no associated with PD which challenges the prioritization quality. However; as can be noted in the functional interaction network (see Fig. 5), many of these genes (represented as blue nodes) have a functional connection with important biological processes or genes directly related with PD (represented as red or green nodes). It has to be mentioned that 10 out of these 22 genes (ACP2, BAI3, CRYZL1, GPR161, HECA, NRXN3, QPCT, SCN3B, TRAPPC2L, VPS4B) has no interactions in this space and therefore are not included in this network and that all disconnected clusters and/or nodes in this network are actually connected through UBC gene as can be confirmed in the full network provided in the supplementary information.

An important finding in this network is that even when PPARGC1A was not identified in our study, several genes were found to be direct interactors, and biological process directly related with this gene are clearly present in our prioritized genes. Specifically, can be confirmed that PPARGC1A is connected through short paths with several of the final 50 genes with reported associations with PD (such as TH, AGTR1 and FABP7) or other without current associations with the disease such as PPM1B or CPT1B. On the other hand, the GO enrichment analysis based on this functional interaction network includes several biological process related with the PPARGC1A function. See details in the supplementary information.

The GO enrichment analysis was conducted (based on DAVID) in order to access to significant biological process encoded by the set of genes in this functional interaction network. Contrary to what was expected due to the risk of the “dopamine bias”, from this analysis is clear the highly significant role of RNA splicing [103] (through several mechanisms) and energy metabolism [46–49, 53] compared with the dopamine metabolism process. This last, although statistically significant was placed well below the two former biological process which on the other hand, have been well associated to PD and unrelated to dopamine metabolism. Again, this suggests that the consensus strategy proposed in this work is not affected by the dopamine bias.

***Dopamine Bias****.* As declared from the beginning, the dopamine bias was considered in the discussion of every prioritization method applied. A last experiment was expressly conducted to evaluate this important issue. For this, a functional interaction network was constructed with the aid of STRING from the set of 246 unique genes coming from the union of ML and *Limma* prioritizations (see Fig. 5).

If we look for those genes in the KEGG Dopaminergic Synapse Pathway (129 genes in the DA Pathway) and in the KEGG Parkinson’s Disease Pathway (142 genes in the PD Pathway) comprised in the set of 246 unique genes coming from the union of ML and *Limma* prioritizations, it is possible to note that only 4.47 % (11 DA genes out of 246) of this set corresponds to the DA pathway, which indicates an insignificant risk of “dopamine bias” for this set. If we also consider that four out of this eleven DA genes are involved in the PD pathway such risk becomes really insignificant. More importantly, the set of 56 genes shared by ML and *Limma* prioritizations only involves five (DRD2, TH, SLC6A3 and SLC18A2) out of the 129 genes in the KEGG DA pathway. Only one (ITPR1) of these five genes was exclusive of the DA pathway, the other four genes were also included in the KEGG PD pathway. This is a clear indicator of the benefits provided by the integration of conceptually different approaches regarding to avoid the “dopamine bias”. All this information can be visually confirmed in the interaction network of genes coming from ML and *Limma* prioritizations provided in Fig. 4. As can be observed in this figure, the ML prioritization is less prone to be affected by the “dopamine bias” which suggest a key role of this approach in reducing such risk.

Finally, only six genes were excluded from the 56 genes from the ML-*Limma* prioritization (CLK1, DDX17, LRP2, NDRG1, SESN1 and SYT17) by concurrently considering the significant PD modules identified in the WGCN analysis (PD_02 and PD_07). Only five out of the 50 prioritized genes were present in the KEGG DA pathway and four out this five dopamine-related genes were included in the KEEG PD pathway. So, from this analysis we can conclude that the consensus strategy proposed in this work is not affected by the “dopamine bias”. See details in Table 11.

**Table 11:** Number of genes in the KEGG DA Pathway, KEGG PD Pathway, and both KEGG DA and PD Pathways in the respective prioritized gene sets

Prioritization Approach	*N*	*n(%)*			*%*
		*DA*	*PD*	*DA-PD*	*DA-PD/DA*
ML ∪ *Limma*	246	11(4.47)	6(2.44)	4(1.63)	36.36
ML	168	7(4.17)	5(2.98)	4(2.38)	57.14
*Limma*	134	9(6.72)	6(4.48)	4(2.99)	44.44
ML ∩ *Limma*	56	5(8.93)	5(8.93)	4(7.14)	80.00
Only-ML	112	2(1.79)	0(0.00)	0(0.00)	0.00
Only-*Limma*	78	4(5.13)	1(1.28)	0(0.00)	0.00
Consensus	50	5(10.00)	5(10.00)	4(8.00)	80.00

*N*: Number of genes prioritized; *n*: number; *%*: percentage; *DA*: genes in the KEGG Dopaminergic Synapse Pathway; *PD*: genes in the KEGG Parkinson’s Disease Pathway; *DA-PD*: genes in the KEGG Dopaminergic Synapse Pathway and the KEGG Parkinson’s Disease Pathway

## Conclusions

A hydrid gene prioritization approach was applied to PD. Specifically, the set of 50 genes prioritized with the proposed consensus strategy was statistically significant, biologically relevant, highly enriched with know PD related genes and exhibited an excelent early recognition ability. In addition to 20 know PD related genes, eight potentially novel PD biomarkers or therapeutic targets (CCNH, DLK1, PCDH8, SLIT1, DLD, PBX1, INSM1, and BMI1) were identified. Additionally, a statistically rigorous approach of standard use in chemoinformatics was proposed to evaluate the early recognition ability of gene prioritization tools. We also demonstrated that the proper combination of several sources of information is a suitable strategy for module prioritization in co-expression networks analysis. Finally, it is possible to assert that the proposed consensus strategy represents an efficient and biologically relevant approach for gene prioritization tasks, providing a valuable decision-making tool for the study of PD pathogenesis and the development of disease-modifying PD therapeutics.

## Additional files

Additional file 1: 1) **Figure S1.** Functional interaction network of the final set of 50 genes prioritized with the consensus strategy and 100 additional interacting genes including UBC. 2) **Table S1.** Samples distribution used for ML analysis. 3) **Table S2.** Sets of PD relevant genes identified by the thirteen ML classification algorithms. 4) **Table S3.** Details on the 50 genes prioritized by means of the proposed consensus strategy. 5) Attribute evaluators used in the consensus ranking analysis. 6) Hypergeometric probability test details. 7) PD related terms in GAD used to identify the set of 513 PD related genes. 8) Composition of the sorted genes lists corresponding to the four prioritization strategies (*Limma*, ML, ML-*Limma*, and Consensus). (DOCX 2196 kb) Additional file 2: Normalized expression values of the 8477 common genes for each of the 102 samples, sample and study identifiers, disease factor (PD or HC), as well as the distribution of training and test samples. (TXT 10084 kb) Additional file 3: Details of the reduced gene set by using the mRMR software. (TXT 54 kb) Additional file 4: Details on the genes sets prioritized by the respective approaches. (TXT 23 kb) Additional file 5: 1) Results of the *Limma* prioritization for the top 1016 genes with uncorrected *p*-values < 0.05. 2) Results of the gene ontology (biological process) enrichment analysis for the top 134 genes prioritized with *Limma* with FDR corrected *p*-values < 0.05. 3) Results of the gene ontology (biological process) enrichment analysis for the top 1016 genes prioritized with *Limma* with uncorrected *p*-values < 0.05. 4) List of the 168 genes prioritized with machine learning. 5) Results of the gene ontology (biological process) enrichment analysis for the 168 genes prioritized with machine learning. 6) Results of the gene ontology (biological process) enrichment analysis for the 1437 genes included in the co-expression module PD_02. 7) Results of the gene ontology (biological process) enrichment analysis for the 494 genes included in the co-expression module PD_07. 8) Results of the ToppCluster combined enrichment analysis for the co-expression modules PD_02 and PD_07. 9) Results of the gene ontology (biological process) enrichment analysis for the 50 genes prioritized with the consensus strategy and 100 additional interacting genes included in the STRING functional interaction network. (XLSX 493 kb)

## Abbreviations

PD: parkinson’s disease
ML: machine learning
WGCN: weighted genes co-expression network
GO: gene ontology
*AUAC*: area under the accumulation curve
*ROC*: area under the ROC curve
*BEDROC*: Boltzmann-enhanced discrimination of ROC
*EF*: enrichment factor
*RIE*: robust initial enhancement
TP: true positives
FP: false positives
DA: dopamine
PPI: prepulse inhibition
