# Supplementary material for: Efficient and biologically relevant consensus strategy for Parkinson’s disease gene prioritization
Source: BMC Med Genomics. 2016 Mar 9;9:12. doi: 10.1186/s12920-016-0173-x (PMC4784386; doi:10.1186/s12920-016-0173-x)
Supplement: Additional file 1: — 1) Figure S1. Functional interaction network of the final set of 50 genes prioritized with the consensus strategy and 100 additional interacting genes including UBC. 2) Table S1. Samples distribution used for ML analysis. 3) Table S2. Sets of PD relevant genes identified by the thirteen ML classification algorithms. 4) Table S3. Details on the 50 genes prioritized by means of the proposed consensus strategy. 5) Attribute evaluators used in the consensus ranking analysis. 6) Hypergeometric probability test details. 7) PD related terms in GAD used to identify the set of 513 PD related genes. 8) Composition of the sorted genes lists corresponding to the four prioritization strategies (Limma, ML, ML-Limma, and Consensus). (DOCX 2196 kb) [file 12920_2016_173_MOESM1_ESM.docx]

**SUPPLEMENTARY INFORMATION**

Efficient and biologically relevant consensus strategy for Parkinson´s disease gene prioritization

Maykel Cruz-Monteagudo^1,2^, Fernanda Borges^1,^*, Cesar Paz-y-Miño^2^, M. Natália D. S. Cordeiro^3^, Irene Rebelo^4^, Yunierkis Perez-Castillo^5,6^, Aliuska Morales Helguera^6^, Aminael Sánchez-Rodríguez^7,^* and Eduardo Tejera^2^

^1^ CIQUP/Departamento de Química e Bioquímica, Faculdade de Ciências, Universidade do Porto, Porto 4169-007, Portugal.

^2^ Instituto de Investigaciones Biomédicas (IIB), Universidad de Las Américas, 170513 Quito, Ecuador.

^3^ REQUIMTE, Department of Chemistry and Biochemistry, Faculty of Sciences, University of Porto, 4169-007 Porto, Portugal.

^4^ REQUIMTE, Department of Biochemistry, Faculty of Pharmacy, University of Porto, 4050-313 Porto, Portugal.

^5^ Sección Físico Química y Matemáticas, Departamento de Química, Universidad Técnica Particular de Loja, San Cayetano Alto S/N, EC1101608 Loja, Ecuador.

^6^ Molecular Simulation and Drug Design Group, Centro de Bioactivos Químicos (CBQ), Central University of Las Villas, Santa Clara, 54830, Cuba.

^7^ Departamento de Ciencias Naturales, Universidad Técnica Particular de Loja, Calle París S/N, EC1101608 Loja, Ecuador.

**E-mail Addresses:** Maykel Cruz-Monteagudo ([gmailkelcm@yahoo.es](mailto:gmailkelcm@yahoo.es)), Fernanda Borges ([fborges@fc.up.pt](mailto:fborges@fc.up.pt)), Cesar Paz-y-Miño ([cesar.pazymino@udla.edu.ec](mailto:cesar.pazymino@udla.edu.ec)), M. Natália D. S. Cordeiro ([ncordeir@fc.up.pt](mailto:ncordeir@fc.up.pt)), Irene Rebelo ([irebelo@ff.up.pt](mailto:irebelo@ff.up.pt)), Yunierkis Perez-Castillo ([yunierkis@gmail.com](mailto:yunierkis@gmail.com)), Aliuska Morales Helguera ([aliuska@uclv.edu.cu](mailto:aliuska@uclv.edu.cu)), Aminael Sanchez-Rodriguez ([asanchez2@utpl.edu.ec](https://mail.udla.edu.ec/owa/?ae=Item&t=IPM.Note&a=New&to=asanchez2%40utpl.edu.ec&nm=asanchez2%40utpl.edu.ec)), Eduardo Tejera ([eduardo.tejera@udla.edu.ec](mailto:eduardo.tejera@udla.edu.ec))

*** Corresponding Authors:** Fernanda Borges [fborges@fc.up.pt](mailto:fborges@fc.up.pt) - Aminael Sanchez-Rodriguez [asanchez2@utpl.edu.ec](https://mail.udla.edu.ec/owa/?ae=Item&t=IPM.Note&a=New&to=asanchez2%40utpl.edu.ec&nm=asanchez2%40utpl.edu.ec)

**CONTENT**

- **Figure S1.** Functional interaction network of the final set of 50 genes prioritized with the consensus strategy and 100 additional interacting genes including UBC.
- **Table S1.** Samples distribution used for ML analysis.
- **Table S2**. Sets of PD relevant genes identified by the thirteen ML classification algorithms.
- **Table S3**. Details on the 50 genes prioritized by means of the proposed consensus strategy.
- **Attribute evaluators used in the consensus ranking analysis.**
- **Hypergeometric probability test details.**
- **PD related terms in GAD used to identify the set of 513 PD related genes.**
- **Composition of the sorted genes lists corresponding to the four prioritization strategies (Limma, ML, ML-Limma, and Consensus).**

**
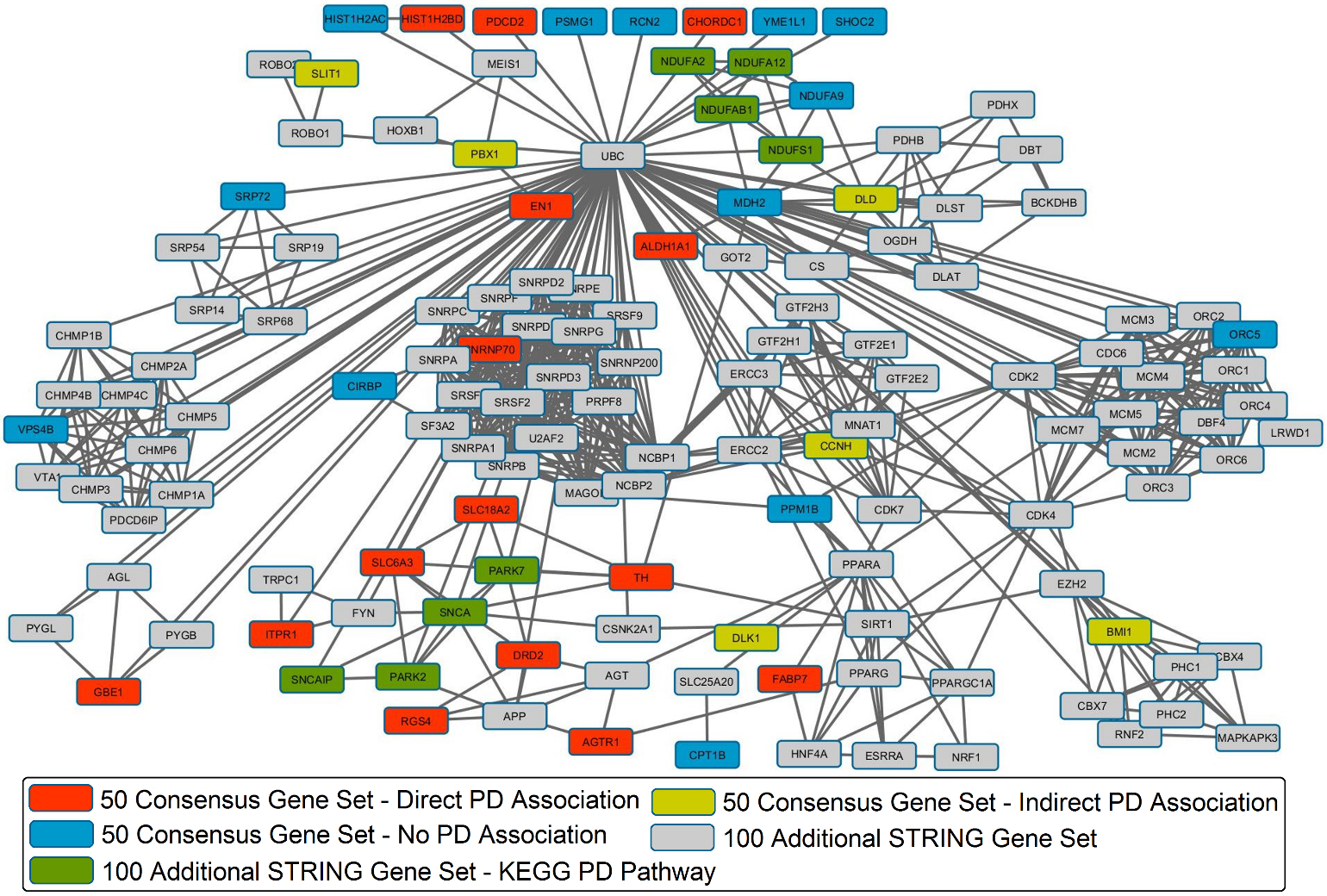
**

**Figure S1.** Functional interaction network of the final set of 50 genes prioritized with the consensus strategy and 100 additional interacting genes including UBC. Each gene node was labeled in order to differentiate those genes in the 50 genes prioritized with the consensus strategy from the 100 additional interacting genes (labeled in gray). Genes with direct, indirect and no literature evidences of association with PD among the 50 genes prioritized with the consensus strategy were labeled in red, yellow and blue, respectively. Those genes among the 100 additional interacting genes included in the KEGG PD pathway were labeled in green.

**Table S1.** Samples distribution used for ML analysis.

| **Study Code** | **Training Set** | | **Test Set** | |
| --- | --- | --- | --- | --- |
|  | ***PD*** | ***HC*** | ***PD*** | ***HC*** |
| GSE20292 | 8 | 11 | 3 | 4 |
| GSE7621 | 12 | 7 | 4 | 2 |
| GSE20333 | 4 | 4 | 2 | 2 |
| GSE8397 | 19 | 10 | 7 | 3 |
| *TOTAL* | *43* | *32* | *16* | *11* |

**Table S2**. Sets of PD relevant genes identified by the thirteen ML classification algorithms.

| **ML Classification Algorithm** | **GENES SUBSET (ENTREZ_GENE_ID)** |
| --- | --- |
| *functions.SimpleLogistic* | SLC18A2; PDCD2; USH2A; CCNH; GPR161; SCN3B; HECA; SELPLG; PF4V1; SIRT4; ZMYM5; CHEK1; DRD5; RLBP1; PLOD1; ABCB9 |
| *rules.MODLEM* | AGTR1; PAICS; CRIM1; TSPAN2 |
| *rules.PART* | AGTR1; PAICS; TSPAN2 |
| *trees.ADTree* | AGTR1; SLC18A2; PDCD2; ROR2; PAICS; CRIM1; TSPAN2; MASP2; SLC22A8 |
| *trees.BFTree* | AGTR1; PAICS; TSPAN2 |
| *trees.FT* | SLC18A2; PDCD2; USH2A; CCNH; HECA; PCDHB8; SELPLG; PF4V1; SIRT4; DRD5; PLOD1; ABCB9 |
| *trees.LADTree* | AGTR1; SLC18A2; PDCD2; USH2A; CCNH; SLC6A3; ALDH1A1; ROR2; GPR161; GBE1; NRXN3; PAICS; BMI1; ATP2A3; CRIM1; INSM1; REG3A; MDH2; SELPLG; GNGT1; SHOC2; TSPAN2; SIRT4; LRRC6; BMPR2; PKD10; C11ORF58; RAD21; E2F5; GMCL1 |
| *trees.LMT* | SLC18A2; PDCD2; USH2A; CCNH; HECA; PCDHB8; SELPLG; PF4V1; SIRT4; DRD5; PLOD1 |
| *trees.SimpleCart* | AGTR1; PAICS; TSPAN2 |
| *meta.AdaBoostM1* | AGTR1; SLC18A2; PDCD2; USH2A; CCNH; SLC6A3; ALDH1A1; ROR2; GPR161; GBE1; TH; ORC5; HECA; CACNA2D2; SLIT1; BMI1; NPPC; QPCT; PBX1; CRIM1; CLK1; CIRBP; PDK4; REG3A; PNMT; FABP7; DDX17; DLD; SRP72; CHORDC1; TAZ; TMOD3; PPM1B; PCDHB8; SELPLG; NDUFA9; GNGT1; SESN1; LRP2; SIRT4; MASP2; RCN2; ACP2; KERA; TLL1; ZMYM5; FBLN5; CHEK1; PDE6C; ITGA7; LMAN2; VBP1; SS18L2; DRD5; VRK3; FKBP1B; SCN1B; HIGD1B; LRRC6; BMPR2; LILRA1; CYP39A1; RLBP1; PTPRJ; IVNS1ABP; PKD1; VPS4B; H2BFS; FKSG2; EIF4EBP2; NRG1; IL15; NNT; PIK3CG; PSG1; SPTLC2; SLC22A8; GALT; TMPRSS5; P4HA1; RUNX3; PEX3; MSTN; DAZ1; CDH19; C1ORF21; GOLGA1; F8 |
| *meta.AttributeSelectedClassifier* | AGTR1; ROR2; QPCT |
| *meta.ClassificationViaRegression* | AGTR1; SLC18A2; SLC6A3; ATP2A3 |
| *meta.Decorate* | AGTR1; SLC18A2; PDCD2; USH2A; CCNH; SLC6A3; ALDH1A1; ROR2; GPR161; SCN3B; TH; NRXN3; ORC5; CACNA2D2; SLIT1; PAICS; PCDH8; NPPC; HIST1H2BD; EN1; DLK1; CIRBP; TRIM36; PTPRN2; NDRG1; VWA5A; DRD2; AZGP1; BAI3; CACNB3; PSMG1; TMOD3; MT1G; CHGB; SELPLG; TRAPPC2L; YME1L1; ITPR1; HIST1H2AC; RGS4; TSPAN2; MASP2; CPT1B; SC5DL; TLL1; COL5A3; SLC25A32; CYP27A1; FBLN5; SYT17; ESRRA; CXXC1; PRELP; ITGA7; VAT1; VBP1; CHST3; SYCP1; ZFP37; LRRC6; MMD; FKTN; HK1; IARS; CRYZL1; TMEM5; EIF4EBP2; AHSA1; IL15; BPNT1; ACTG1; COPS3; BAGE; RBL1; STRAP; SNRNP70; DAPK1; RFX3; DIABLO; FOXO4; LOCLOC399491; ABCB9; TLR6; SCNN1A; NFYB; SMPX; IGSF1; POLG; RPS29; SPTAN1; GARS; PRDX6; APC; ATG12; RPL7 |

**Table S3**. Details on the 50 genes prioritized by means of the proposed consensus strategy.

| **Oficial**  **Gene symbol** | **DAVID Gene Name** | **ML**  **Rank** | **LIMMA**  **Rank** | **WGCN**  **Module** | **GAD**  **PD** | **Literature**  **PD** |
| --- | --- | --- | --- | --- | --- | --- |
| SLC18A2 | *solute carrier family 18 (vesicular monoamine), member 2* | 2 | 1 | PD_02 | 1 | 1 |
| AGTR1 | *angiotensin II receptor, type 1* | 1 | 3 | PD_02 | 0 | 1 |
| GBE1 | *glucan (1,4-alpha-), branching enzyme 1* | 10 | 2 | PD_02 | 0 | 1 |
| PDCD2 | *programmed cell death 2* | 3 | 22 | PD_02 | 0 | 1 |
| ALDH1A1 | *aldehyde dehydrogenase 1 family, member A1* | 7 | 5 | PD_02 | 0 | 1 |
| CCNH | *cyclin H* | 5 | 10 | PD_02 | 0 | 0 |
| NRXN3 | *neurexin 3* | 13 | 6 | PD_02 | 0 | 0 |
| SLC6A3 | *solute carrier family 6 (neurotransmitter transporter, dopamine), member 3* | 6 | 17 | PD_02 | 1 | 1 |
| DLK1 | *delta-like 1 homolog (Drosophila)* | 30 | 8 | PD_02 | 0 | 0 |
| GPR161 | *G protein-coupled receptor 161* | 9 | 32 | PD_02 | 0 | 0 |
| SCN3B | *sodium channel, voltage-gated, type III, beta* | 11 | 15 | PD_02 | 0 | 0 |
| TH | *tyrosine hydroxylase* | 12 | 14 | PD_02 | 1 | 1 |
| PCDH8 | *protocadherin 8* | 20 | 12 | PD_02 | 0 | 0 |
| ORC5 | *origin recognition complex, subunit 5-like (yeast)* | 14 | 54 | PD_02 | 0 | 0 |
| HECA | *headcase homolog (Drosophila)* | 15 | 89 | PD_07 | 0 | 0 |
| SLIT1 | *slit homolog 1 (Drosophila)* | 17 | 27 | PD_02 | 0 | 0 |
| BMI1 | *BMI1 polycomb ring finger oncogene* | 19 | 18 | PD_07 | 0 | 0 |
| QPCT | *glutaminyl-peptide cyclotransferase* | 22 | 81 | PD_02 | 0 | 0 |
| DLD | *dihydrolipoamide dehydrogenase* | 41 | 23 | PD_02 | 0 | 0 |
| HIST1H2BD | *histone cluster 1, H2bd* | 24 | 103 | PD_07 | 0 | 1 |
| PBX1 | *pre-B-cell leukemia homeobox 1* | 25 | 34 | PD_02 | 0 | 0 |
| SRP72 | *signal recognition particle 72kDa* | 46 | 25 | PD_02 | 0 | 0 |
| DRD2 | *dopamine receptor D2* | 43 | 26 | PD_02 | 1 | 1 |
| EN1 | *engrailed homeobox 1* | 28 | 86 | PD_02 | 1 | 1 |
| TRIM36 | *tripartite motif-containing 36* | 34 | 29 | PD_02 | 0 | 1 |
| INSM1 | *insulinoma-associated 1* | 29 | 40 | PD_02 | 0 | 0 |
| MDH2 | *malate dehydrogenase 2, NAD (mitochondrial)* | 42 | 30 | PD_02 | 0 | 0 |
| CIRBP | *cold inducible RNA binding protein* | 31 | 85 | PD_02 | 0 | 0 |
| FABP7 | *fatty acid binding protein 7, brain* | 36 | 37 | PD_02 | 0 | 1 |
| PTPRN2 | *protein tyrosine phosphatase, receptor type, N polypeptide 2* | 37 | 58 | PD_02 | 0 | 1 |
| PSMG1 | *proteasome (prosome, macropain) assembly chaperone 1* | 50 | 39 | PD_02 | 0 | 0 |
| VWA5A | *von Willebrand factor A domain containing 5A* | 40 | 48 | PD_02 | 1 | 1 |
| ITPR1 | *inositol 1,4,5-triphosphate receptor, type 1* | 61 | 42 | PD_02 | 0 | 1 |
| BAI3 | *brain-specific angiogenesis inhibitor 3* | 45 | 61 | PD_02 | 0 | 0 |
| CPT1B | *choline kinase beta; carnitine palmitoyltransferase 1B (muscle)* | 72 | 46 | PD_07 | 0 | 0 |
| CACNB3 | *calcium channel, voltage-dependent, beta 3 subunit* | 47 | 66 | PD_02 | 0 | 0 |
| ACP2 | *acid phosphatase 2, lysosomal* | 73 | 47 | PD_02 | 0 | 0 |
| CHORDC1 | *cysteine and histidine-rich domain (CHORD)-containing 1; cysteine and histidine-rich domain (CHORD)-containing 1 pseudogene* | 48 | 74 | PD_07 | 1 | 1 |
| SHOC2 | *soc-2 suppressor of clear homolog (C. elegans)* | 65 | 49 | PD_02 | 0 | 0 |
| VBP1 | *von Hippel-Lindau binding protein 1* | 91 | 52 | PD_02 | 0 | 0 |
| PPM1B | *protein phosphatase 1B (formerly 2C), magnesium-dependent, beta isoform* | 52 | 108 | PD_07 | 0 | 0 |
| YME1L1 | *YME1-like 1 (S. cerevisiae)* | 59 | 56 | PD_02 | 0 | 0 |
| NDUFA9 | *NADH dehydrogenase (ubiquinone) 1 alpha subcomplex, 9, 39kDa* | 57 | 62 | PD_02 | 0 | 0 |
| TRAPPC2L | *trafficking protein particle complex 2-like* | 58 | 83 | PD_02 | 0 | 0 |
| HIST1H2AC | *histone cluster 1, H2ac* | 62 | 94 | PD_07 | 0 | 0 |
| RGS4 | *regulator of G-protein signaling 4* | 64 | 104 | PD_02 | 0 | 1 |
| CRYZL1 | *crystallin, zeta (quinone reductase)-like 1* | 108 | 71 | PD_02 | 0 | 0 |
| RCN2 | *reticulocalbin 2, EF-hand calcium binding domain* | 71 | 113 | PD_02 | 0 | 0 |
| SNRNP70 | *small nuclear ribonucleoprotein 70kDa (U1)* | 139 | 87 | PD_07 | 0 | 1 |
| VPS4B | *vacuolar protein sorting 4 homolog B (S. cerevisiae)* | 114 | 100 | PD_02 | 0 | 0 |

*ML/Limma Rank*: ranking corresponding to the respective gene according to the *ML*/*Limma* analysis; *WGCN Module*: WGCN PD module including the respective gene; *GAD PD*: “1” means that the respective gene was found on the set of 319 know PD related genes in GAD, and “0” otherwise; *Literature PD*: “1” means that direct evidence was found for the respective gene on an exhaustive literature search for associations between the respective gene and PD, and “0” otherwise.

**Attribute evaluators used in the consensus ranking analysis:**

1. *ChiSquaredAttributeEval*
2. *ClassifierAttributeEval*
3. *CorrelationAttributeEval*
4. *CVAttributeEval*
5. *FilteredAttributeEval*
6. *GainRatioAttributeEval*
7. *InfoGainAttributeEval*
8. *OneRAttributeEval*
9. *ReliefFAttributeEval*
10. *SignificanceAttributeEval*
11. *SymmetricalUncertAttributeEval*)

**Hypergeometric probability test details:**

Unique *Homo sapiens* (human) genes in GAD associated with PD were used to validate those genes sets potentially related with PD identified by our approach. Specifically, 553 unique genes including PD related terms were identified in GAD but only 319 were present in the background of 8477 genes. So, the number of common genes in the genes sets identified by our approach as related with PD, and the set of 319 known PD related genes in GAD was used to evaluate de enrichment capability of our approach by using the *phyper* function of the *R* package *stats* [^1^](#_ENREF_1). Four parameters are considered here: *N* (total gene number of the background used = 8477), *M* (number of known PD related genes in GAD included in the background = 319), *n* (number of prioritized genes), and *m* (number of common genes in *n* and *M*).

**PD related terms in GAD used to identify the set of 513 PD related genes**:

1. *Parkinson*
2. *Parkinson's disease*
3. *Parkinsons disease*
4. *Parkinson disease*
5. *Parkinsonism*
6. *Parkinsonian disorders*
7. *Parkinson disease, secondary*
8. *Depression in parkinson's disease*
9. *Dystonia, acute parkinsonism tardive dyskinesia*

**Composition of the sorted genes lists corresponding to the four prioritization strategies (Limma, ML, ML-Limma, and Consensus):**

Specifically, for the *Limma* strategy, the 8477 genes are increasingly sorted according to the respective fdr adjusted p-values. The ML list is sorted in three successive fractions. The genes in the initial ML fraction are those 168 prioritized genes and are sorted according to the same ranking procedure above described. The second ML fraction is composed by those 332 genes remaining from those 500 selected with the mRMR procedure and sorted by the entropy score used by the mRMR procedure to rank the genes in order of relevance to the factor disease. The last ML fraction inlcudes the remaining 7977 genes which are randomized by using *List Randomizer*, a randomization tool implemmented in [^2^](#_ENREF_2). The first fraction of the ML-*Limma* list comprise 57 common genes prioritized by *Limma* and ML strategies and are sorted by using the *Min-* and *Mean-Rank* fusion rules above described. The second ML-*Limma* fraction is composed by 189 unique genes prioritized by *Limma* and ML strategies which are randomized as above described. The last ML-*Limma* fraction inlcudes the remaining randomized set of 8231 genes. Finally, the initial fraction of the Consensus list is determined by the 50 genes prioritized sorted as previously described. The next Consensus fraction is composed by those genes remaining from the *Limma* and ML strategies and the WGCN modules PD_02 and PD_07, which are randomized. The last Consensus fraction inlcudes the remaining randomized set of 6465 genes.

**REFERENCES**

1. N. L. Johnson, S. Kotz and A. W. Kemp, *Univariate Discrete Distributions*, Wiley, New York, Second edn., 1992.

2. Random.org, List Randomizer, https://[www.random.org/lists/](http://www.random.org/lists/), (accessed March 02, 2015, 2015).
